# Triplet bismuthinidenes featuring unprecedented giant and positive zero field splittings

**DOI:** 10.1093/nsr/nwad169

**Published:** 2023-06-12

**Authors:** Mengyuan Wu, Wang Chen, Dongmin Wang, Yizhen Chen, Shengfa Ye, Gengwen Tan

**Affiliations:** Innovation Center for Chemical Sciences, Key Laboratory of Organic Synthesis of Jiangsu Province, College of Chemistry, Chemical Engineering and Materials Science, Soochow University, Suzhou 215123, China; State Key Laboratory of Catalysis, Dalian Institute of Chemical Physics, Chinese Academy of Sciences, Dalian 116023, China; University of Chinese Academy of Sciences, Beijing 100049, China; Key Laboratory of Bioinorganic and Synthetic Chemistry of Ministry of Education, School of Chemistry, IGCME, Sun Yat-sen University, Guangzhou 510275, China; Innovation Center for Chemical Sciences, Key Laboratory of Organic Synthesis of Jiangsu Province, College of Chemistry, Chemical Engineering and Materials Science, Soochow University, Suzhou 215123, China; Key Laboratory of Bioinorganic and Synthetic Chemistry of Ministry of Education, School of Chemistry, IGCME, Sun Yat-sen University, Guangzhou 510275, China; Innovation Center for Chemical Sciences, Key Laboratory of Organic Synthesis of Jiangsu Province, College of Chemistry, Chemical Engineering and Materials Science, Soochow University, Suzhou 215123, China; State Key Laboratory of Catalysis, Dalian Institute of Chemical Physics, Chinese Academy of Sciences, Dalian 116023, China; Key Laboratory of Bioinorganic and Synthetic Chemistry of Ministry of Education, School of Chemistry, IGCME, Sun Yat-sen University, Guangzhou 510275, China; Innovation Center for Chemical Sciences, Key Laboratory of Organic Synthesis of Jiangsu Province, College of Chemistry, Chemical Engineering and Materials Science, Soochow University, Suzhou 215123, China; State Key Laboratory of Elemento-Organic Chemistry, Nankai University, Tianjin 300071, China

**Keywords:** bismuth, bismuthinidene, triplet state, zero field splitting, theoretical calculation

## Abstract

Isolation of triplet pnictinidenes, which bear two unpaired electrons at the pnictogen centers, has long been a great challenge due to their intrinsic high reactivity. Herein, we report the syntheses and characterizations of two bismuthinidenes M^s^Fluind*^t^*^Bu^-Bi (**3**) and M^s^Fluind*-Bi (**4**) stabilized by sterically encumbered hydrindacene ligands. They were facilely prepared through reductions of the corresponding dichloride precursors with 2 molar equivalents of potassium graphite. The structural analyses revealed that **3** and **4** contain a one-coordinate bismuth atom supported by a Bi–C single σ bond. As a consequence, the remaining two Bi 6p orbitals are nearly degenerate, and **3** and **4** possess triplet ground states. Experimental characterizations with multinuclear magnetic resonance, magnetometry and near infrared spectroscopy coupled to wavefunction based *ab initio* calculations concurred to evidence that there exist giant and positive zero field splittings (>4300 cm^–1^) in their *S* = 1 ground states. Hence even at room temperature the systems almost exclusively populate the lowest-energy nonmagnetic *Ms* = 0 level, which renders them seemingly diamagnetic.

## Teaser

Two triplet bismuthinidenes have been isolated and structurally characterized, but both behave as if they possessed diamagnetic ground states in magnetometric measurements due to the exceedingly strong relativistic effect.

## INTRODUCTION

**Figure 1.**
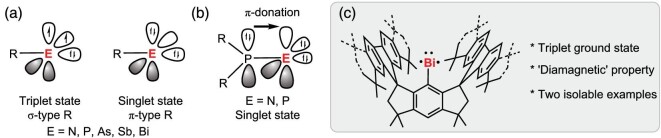
(a) Possible electronic structures of nitrenes and pnictinidenes. (b) The strategy used to stabilize singlet nitrene [14] and phosphinidene [13] reported by Bertrand *et al.* (c) An isolable bismuthinidenes reported in this work.

Low-valent main group compounds are intriguing species thanks to their unique physical and chemical properties [1–3]. Apart from being often used as supporting ligands in transition metal chemistry, they have been shown to play important roles in small molecule activation and catalysis [1–3]. Among them, pnictinidenes R-E (E = P, As, Sb or Bi; R = monoanionic ligands), a group of 15 compounds containing a one-coordinate central atom in the oxidation state of + 1, have attracted a great deal of attention, which is ascribed to their interesting electronic structures and significant uses in synthetic chemistry [4]. They can adopt either a singlet or a triplet electronic ground state, depending on the nature of the attached substituent R (Fig. 1a, where R is an anionic monosubstituted ligand) [5]. The preparation of free pnictinidenes is, however, highly challenging, which lies in the fact such species have a high propensity to undergo self-aggregation leading to dipnictenes or higher oligomers [6–12]. To date, only a limited number of free pnictinidenes have been isolated under standard experimental conditions. A singlet phosphinidene reported by Bertrand and coworkers in 2016 was kinetically stabilized by a bulky π-type diaminophosphino ligand [13], and the same group used a similar approach to access a singlet nitrene [14]. The stabilization of both species was found to be largely accomplished by the π-donation from the phosphinyl center to an empty p orbital at the terminal nitrogen or phosphorus center (Fig. 1b). It is noteworthy that carbyne anions [15] are isoelectronic to pnictinidenes, and a singlet copper(I) carbyne anion complex has recently been reported by Liu *et al.* [16]. In 2020, a transient triplet metallonitrene was characterized by Schneider, Holthausen *et al.* through *in situ* X-ray crystallography at low temperatures, but its high thermal sensitivity hinders the isolation [17].

In comparison to nitrenes and phosphinidenes, the isolation of heavier pnictinidenes is more difficult attributed to more diverging *ns* and *np* orbitals when descending the periodic table of elements [18]. Therefore, stibinidenes and bismuthinidenes are typically stabilized by coordination with Lewis bases leading to two- or three-coordinate metal centers, which, however, significantly changes their ground-state electronic structures from a triplet to a singlet [19–23]. Cornella *et al*. showed that chelating N, C, N-ligated bismuthinidenes could act as efficient catalysts through Bi(I)/Bi(III) redox couples [24–26].

Triplet bismuthinidenes, BiX (X = H, F, Cl, Br, I, Me and AlCl_4_), have been studied in the gas phase at elevated temperatures (> 400 K) since the 1960s [27–29]. Very recently, using our own developed sterically encumbered hydrindacene ligands, we succeeded in accessing base-stabilized germylidenylpnictinidenes [30], free germylyne [31] and stannylyne radicals [32], and more strikingly, a triplet stibinidene [33]. As our ongoing research targeted heavier free pnictinidenes, here we report the syntheses, characterizations and reactivity studies of two highly thermally stable bismuthinidenes, **3** and **4** (Fig. 1c). However, during the preparation of this manuscript, exactly the same compound **3** was reported by Cornella and coworkers [34]. Combined spectroscopic and computational studies demonstrate that **3** and **4** feature triplet electronic ground states with the two unpaired electrons dominantly residing in the two energetically near-degenerate Bi 6p_x_ and 6p_y_ orbitals. Remarkably, spectroscopic and magnetometric measurements revealed that their paramagnetic ground states feature gigantic, positive zero-field splittings (>4300 cm^–1^), which render them seemingly diamagnetic up to room temperature.

## RESULTS AND DISCUSSION

**Scheme 1.**
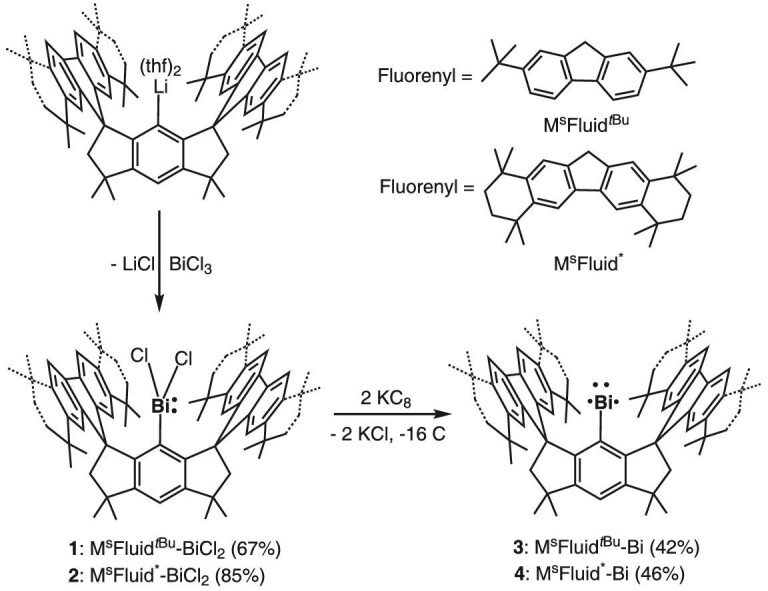
Syntheses of bismuthinidenes through the reductions of Bi(III) precursors with potassium graphite.

The Bi(III) precursors M^s^Fluind*^t^*^Bu^-BiCl_2_ (**1**) and M^s^Fluind*-BiCl_2_ (**2**) were readily synthesized through the reactions of the corresponding lithium salts and bismuth trichloride in THF at room temperature (Scheme 1). Compounds **1** and **2** were isolated in moderate yields as colorless solids. The molecular structures determined by single-crystal X-ray diffraction (SC-XRD) analyses are depicted in Figures S1 and S2 in Supplementary Information (SI), respectively. We then carried out the reduction reactions of **1** and **2** in THF with 2 molar equivalents of KC_8_ in THF at room temperature. Upon workup, bismuthinidenes **3** and **4** were isolated as yellow crystals in 42% and 46% yields, respectively. Compound **3** is marginally soluble in *n*-hexane, but moderately soluble in toluene and benzene, while **4** has a good solubility in all these solvents. Although highly air-sensitive, they can be kept at room temperature under a N_2_ atmosphere for at least 1 month, and can even be heated to 80^o^C for 6 hours in benzene solutions without noticeable decomposition.

**Figure 2.**
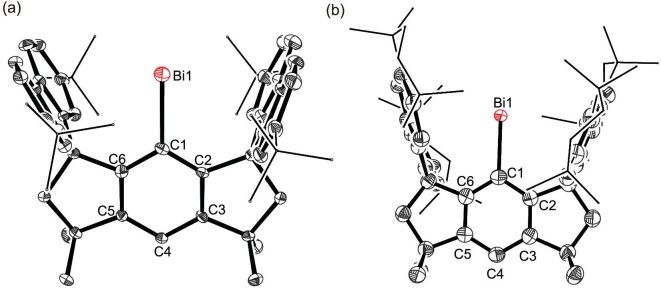
Thermal ellipsoid drawings of the molecular structures of **3** (a) and **4** (b). All hydrogen atoms are omitted, and the substituents at the fluorenyl moieties are shown in a wireframe style for clarity. Selected bond lengths (Å) and angles (^o^): **3**: Bi1–C1 2.284(4), C1–C2 1.397(5), C1–C6 1.408(5), C2–C3 1.394(5), C3–C4 1.399(5), C4–C5 1.376(5), C5–C6 1.404(5); C2-C1-Bi1 121.6(3), C6-C1-Bi1 121.3(3). **4**: Bi1–C1 2.275(3), C1–C2 1.399(5), C1–C6 1.398(5), C2–C3 1.397(5), C3–C4 1.388(5), C4–C5 1.390(5), C5–C6 1.402(5); C2-C1-Bi1 121.8(2), C6-C1-Bi1 122.0(2).

Molecular structures of **3** and **4** were unambiguously determined by SC-XRD analyses (Fig. 2). Both crystallize in the triclinic space group *P*-1, and feature similar geometric parameters; therefore, we mainly discuss those of **3** here. The Bi atom is only bonded to the C atom of the ligand central phenyl (Ph) ring with a Bi–C distance of 2.284(4)  Å, which is comparable to those in **1** (2.278(6) Å) and a dibismuthene (2.290(7)  Å) reported by Tokitoh *et al.* [9], indicative of a single bond character. The Bi atom resides at the center of the ligand pocket as suggested by the almost identical angles of Bi1-C1-C6 (121.3(3)^o^) and Bi1-C1-C2 (121.6(3)^o^). Moreover, the shortest distance of ∼3.4 Å from the Bi atom to the flanking fluorenyl substituents in **3** indicates the absence of noticeable covalent bonding interactions between them [35], but the presence of van der Waals interactions [36,37]. The two neighboring molecules of **3** exhibit a face-to-face interaction with an intermolecular Bi•••Bi distance of 4.405 Å (Supplementary Figure S3). This long distance, which substantially exceeds the Bi–Bi single bond length in {[(SiMe_3_)_2_CH]_2_Bi}_2_ (3.0053(4)  Å) [38], and, more importantly, the sum of the van der Waals radii of two Bi atoms (4.14 Å) [36,37], evidences that there exists no Bi–Bi bonding interaction. Analogously, the Bi center is well-separated by the ligand skeleton in **4** (Supplementary Figure S4), and the shortest Bi•••Bi distance of 13.627 Å significantly surpasses that in **3**, which is attributed to the presence of two bulkier flanking fluorenyl substituents in **4**. Therefore, **3** and **4** represent the first isolable examples of free bismuthinidenes containing one-coordinated Bi atoms. In fact, the isolation of one-coordinate main-group species remains a formidable task, and very recently one-coordinate Al(I) compounds have been reported by Power *et al*. [39] and Zhang and Liu [40].

**Figure 3.**
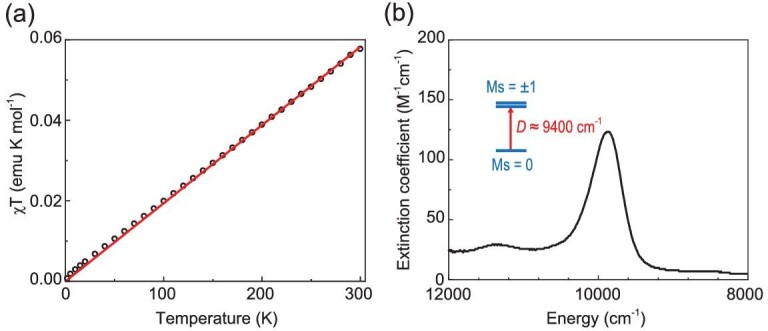
(a) Variable temperature magnetization measurements of a solid sample of **3**. The dots represent experimental measurements and the solid line (red) represents fitting with χ_TIP_ = 194 × 10^−6^ emu. (b) Near infrared absorption spectrum of **3** in THF (1.014 mM) at room temperature. (See online supplementary material for a color version of this figure.)

All features in the ^13^C and ^1^H NMR spectra of **3** and **4** recorded in solutions are in the typical diamagnetic region and similar to those of the Bi(III) precursors, except those for atoms in the vicinity of the Bi center (Supplementary Figures S23–S26). For **3**, the most remarkable feature is that the ^13^C resonance for the carbon connected to the Bi atom has a chemical shift of *δ* –204.8 ppm at room temperature, whereas the corresponding ^13^C signals in **1** and 2,6-Trip_2_C_6_H_3_-BiCl_2_ (Trip = 2,4,6-*i*Pr_3_C_6_H_2_) [8] appear at *δ* 213.0 and 214.6 ppm, respectively. Similarly, an upfield shift was also found for the resonance of the Ph proton at the para-position to the bismuth atom at *δ* –1.06 ppm in **3** relative to *δ* 7.58 ppm observed for **1**. The heteronuclear single quantum correlation (HSQC) spectrum clearly shows the correlation of this signal with the carbon feature at *δ* 160.2 ppm (Supplementary Figure S9). In analogy to **3**, the corresponding C and H atoms in **4** exhibit abnormally upfield shifted NMR signals at *δ* –189.5 and –0.63 ppm in comparison to those at *δ* 219.3 and 7.50 ppm in **2**, respectively. Moreover, the absence of any absorption at around *v* of 1700 cm^–1^ in the infra-red spectrum (Supplementary Figure S10) rules out the possibility that there is any hydride bonded to the bismuth center [41]. Based on all findings, we surmised that **3** and **4** have an *S* = 1 ground state. As integer spin systems are usually silent for conventional X-band EPR spectrometers, we thus carried out variable-temperature (T) magnetic susceptibility (χ) measurements with a superconducting quantum interference device (SQUID). However, as shown in Fig. 3a, the χT value thus obtained varies linearly with respect to T. Consequently, χT can be reasonably modelled by a moderate temperature independent paramagnetism (TIP) term of 194 × 10^−6^ emu for an *S* = 0 ground state, because after subtraction of the TIP, the χT value is essentially independent of T. These findings seem to be incompatible with the ground-state spin multiplicity deduced from multinuclear NMR investigations and necessitates further scrutiny.

**Figure 4.**
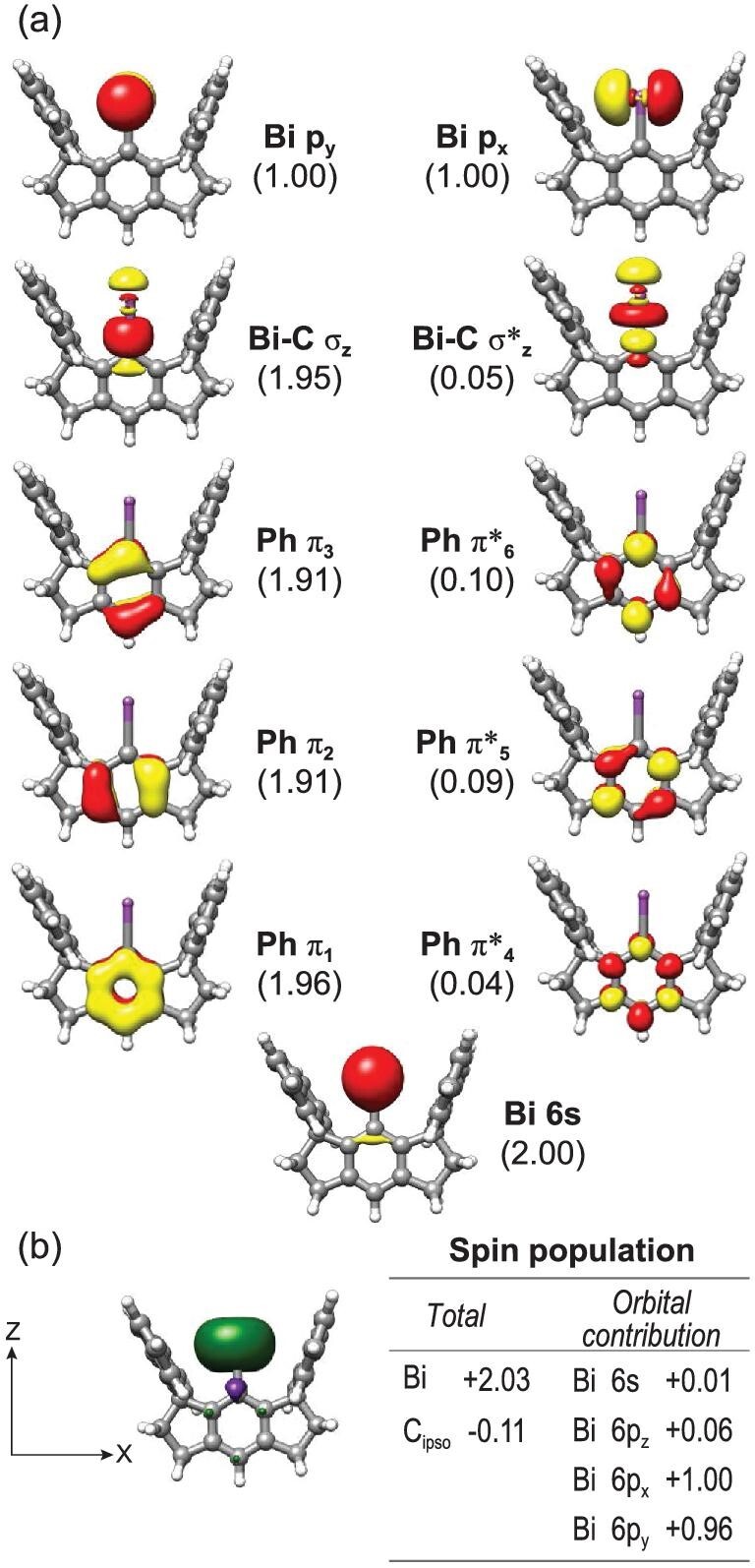
(a) Natural orbitals of the ground-state electronic structure of **3ʹ** obtained from CASSCF(12,11) calculations with the corresponding occupation number in parentheses listed below each orbital label. (b) Computed spin density, spin population and orbital contributions thereof.

To elucidate the electronic structure of bismuthinidene **3**, we undertook detailed theoretical computations. Noncovalent interaction (NCI) [42] analyses at the PBE level of theory employing the scalar relativistic second-order Douglas-Kroll-Hess Hamiltonian [43] (Supplementary Figure S12) show that, except for van der Waals interactions, there is no other considerable bonding between the Bi center and the flanking fluorenyl functionalities. Therefore, we hereafter focus on the covalent bonding between the Bi atom and the central Ph group. To this end, wavefunction based multireference CASSCF [44]/NEVPT2 [45] (CASSCF = the complete active space self-consistent field, NEVPT2 = *N*-electron valence perturbation theory up to the 2nd order) computations were performed, for which a simplified model (**3ʹ**) was employed where all methyl substituents of **3** were replaced by hydrogen atoms. An active space was chosen to distribute 12 electrons into 11 orbitals including Bi–C σ and σ*, Bi 6s and 6p orbitals as well as the six π orbitals of the Ph ring. Theoretical results suggested that **3ʹ** possesses a triplet ground state that is 18.4 and 18.5 kcal/mol lower in energy than the open- and closed-shell singlet states, respectively. As shown in Fig. 4a, the dominant electron configuration of the triplet ground state is (Bi 6s)^2^(Ph π_1,2,3_)^6^(Bi–C σ_z_)^2^(Bi 6p_x_)^1^(Bi 6p_y_)^1^(Ph π*_4,5,6_)^0^(Bi–C σ*_z_)^0^ and accounts for 86% of the wavefunction. Because of the exceedingly large energy separation between Bi 6p and C 2p atomic orbitals, one hardly identifies any discernible π-bonding between Bi 6p_y_ and Ph C 2p orbitals. The Bi–Ph interaction is thus best described as a single σ bond, consistent with the computed Mayer bond order of 0.77. In other words, the Bi 6p_x_ and 6p_y_ orbitals are nearly degenerate. In line with this notion is that both orbitals make nearly identical contributions to the spin population, thereby resulting in donut-like positive spin density around the Bi center (Fig. 4b). As such, **3** ought to have a triplet ground state on the grounds of Hund's rule. On the other hand, if it possessed a diamagnetic ground state with a nominal vacant Bi 6p_y_ orbital, for the same reason discussed above, the π donation of the occupied Ph π orbitals into Bi 6p_y_ would not be strong enough to stabilize such an *S* = 0 ground state.

CASSCF(12,11)/NEVPT2 calculations predicted that **3ʹ** possesses an axial zero-field splitting (ZFS) *D* >4300 cm^–1^ (Supplementary Table S1). Due to such a huge, positive ZFS, the system almost exclusively populates at the lowest-energy non-magnetic *Ms* = 0 level even at room temperature, whereas the populations of the excited magnetic *Ms* = ±1 levels are negligible. As a consequence, the precise ground state of **3** cannot be readily determined by SQUID measurements up to 300 K (Supplementary Figure S14). As elaborated in the Supporting Information, this unprecedented giant *D* value originates from the exceptionally strong spin-orbit coupling (SOC) between the triplet ground state and low-lying closed-shell singlet excited states, which gets accentuated by the effective SOC constant of Bi reaching as high as 12 000 cm^–1^ [46]. Bi_2_^2–^ dianion, which is isoelectronic to triplet dioxygen, also exhibited a diamagnetic nature owing to the strong SOC effect of the Bi atom [47,48]. Consistent with the theoretical prediction, the near infrared spectrum of **3** registers a broad absorption peak at 9870 cm^–1^ with a band width of 570 cm^–1^ (Fig. 3b and Supplementary Figure S11). This feature has a low intensity (ϵ = 120 M^–1^ cm^–1^), which likely reflects its formally spin-forbidden nature; thus, we tentatively attributed it to *Ms* = 0 → *Ms* = ±1 transitions, an assignment that requires further experimental verification.

**Scheme 2.**
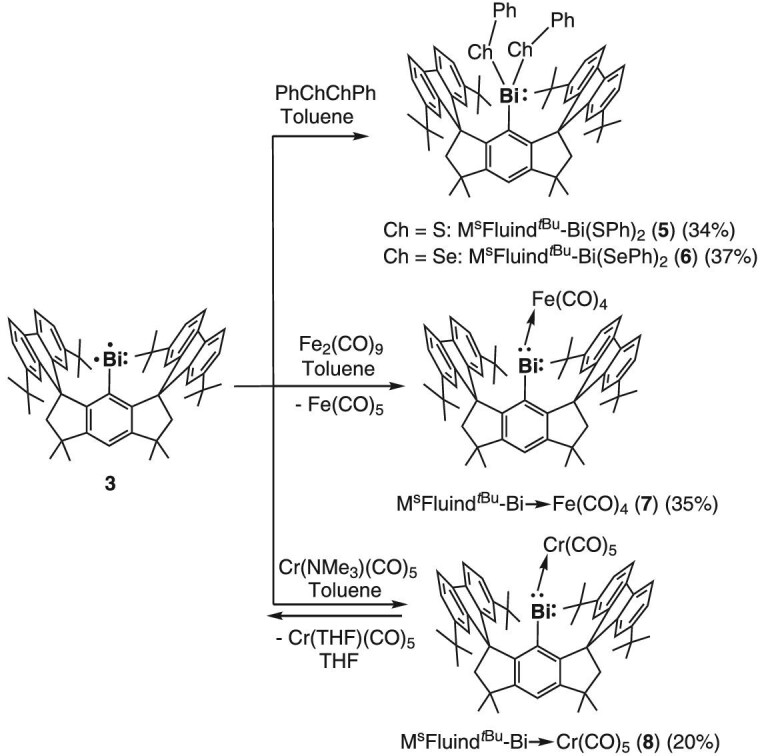
Reactivity studies of **3**.

We further performed reactivity studies of **3** to have a better understanding of its chemical properties. Facile oxidative addition of PhChChPh (Ch' = S and Se) to the Bi center in **3** was observed to afford Bi(III) compounds M^s^Fluid*^t^*^Bu^-Bi(Ch’Ph) (**5**: Ch' = S; **6**: Ch' = Se) (Scheme 2), consistent with the + 1 oxidation state of the Bi atom in **3**. Moreover, the reaction of **3** with PhSSPh in a C_6_D_6_ solution was tested in a NMR tube, but did not show the formation of H_2_, further confirming that no hydride is attached to the Bi atom in **3** (Supplementary Figure S29). The molecular structures of **5** and **6** were unambiguously determined by SC-XRD analyses as shown in Supplementary Figures S5 and S6, respectively.

Upon treatment of **3** with Fe_2_(CO)_9_ and Cr(NMe_3_)(CO)_5_, complexes M^s^Fluid*^t^*^Bu^-Bi→Fe(CO)_4_ (**7**) and M^s^Fluid*^t^*^Bu^-Bi→Cr(CO)_5_ (**8**) were isolated as purple and green crystals, respectively. Interestingly, **7** and **8** feature singlet ground states as shown by NMR spectroscopy. It is noteworthy that the Cr(CO)_5_ unit is only weakly coordinated to the bismuth atom in **8**, and readily dissociates in a THF-D_8_ solution to convert back to **3**. The molecular structures of **7** (Supplementary Figure S7) and **8** (Supplementary Figure S8) show that the Bi–Fe (2.5501(9) Å) and Bi–Cr (2.7263(14) Å) distances are shorter than the corresponding Bi–M bonds (2.6705 and 2.8144(19) Å, respectively) in N, C, N-chelated bismuthinidene-metal carbonyl complexes [49]. This is most probably attributed to the low-coordinate nature of the Bi atoms in **7** and **8**, which strengthens π-back-donation interactions with transition metal centers. To the best of our knowledge, **7** and **8** are the first bismuthinidene complexes containing two-coordinate Bi atoms.

Theoretical calculations predicted that the triplet-singlet gaps of **7** and **8** are as high as 16.3 and 38.9 kcal/mol, respectively, and hence confirmed their diamagnetic ground states. Both systems feature considerable π-back-donation from the transition metal moieties to the unoccupied p_π_ acceptor orbital of the Bi center (Supplementary Figures S15 and S16), an analogous stabilizing effect was also observed for singlet phosphinidene complexes [50]. Upon closer inspection, the π-back-donation in **7** is much stronger than that in **8**, presumably due to the attenuated π donation ability of the Cr(CO)_5_ unit having a relatively electron deficient Cr^0^ center compared to Fe^0^, which accounts for their distinct Bi–Fe and Bi–Cr bond lengths and bonding strengths.

## CONCLUSION

In summary, this work demonstrated that bismuthinidenes **3** and **4** featuring essentially one-coordinate Bi atoms can be isolated in the condensed phase at room temperature by utilizing the sterically congested hydrindacene ligands. Theoretical analyses revealed that the one-coordinate Bi atom features two nearly degenerate Bi 6p_x_ and 6p_y_ orbitals; thus, the bismuthinidenes favor a triplet, instead of a singlet, ground state with a leading electron configuration of (Bi–C σ_z_)^2^(Bi 6p_x_)^1^(Bi 6p_y_)^1^. They represent the first isolable examples of Lewis base-free bismuthinidenes. Multinuclear NMR, SQUID and near infrared measurements revealed that the positive, gigantic ZFSs (>4300 cm^–1^) in their *S* = 1 ground states render them to behave as if they had *S* = 0 ground states. The unique electronic structures of the bismuthinidenes may lead to interesting reactivity, which is currently under investigation in our laboratory.

## ADDITIONAL INFORMATION

X-ray data are available from the Cambridge crystallographic Data Centre under reference numbers CCDC 2227156 (**1**), 2227157 (**2**), 2227158 (**3**), 2227159 (**4**), 2227160 (**5**), 2227161 (**6**), 2227162 (**7**), 2227163 (**8**). These data can be obtained free of charge from the CCDC at http://www.ccdc.cam.ac.uk/data_request/cif. All other experimental, spectroscopic, crystallographic, and computational data are included in the Supplementary Information.

## Supplementary Material

nwad169_Supplemental_File
