# Supplementary material for: Triplet bismuthinidenes featuring unprecedented giant and positive zero field splittings
Source: Natl Sci Rev. 2023 Jun 12;10(10):nwad169. doi: 10.1093/nsr/nwad169 (PMC10684269; doi:10.1093/nsr/nwad169)
Supplement: nwad169_Supplemental_File [file nwad169_supplemental_file.docx]

**Supplementary Information for**

Triplet Bismuthinidenes Featuring Unprecedented Giant and Positive Zero Field Splittings

By Mengyuan Wu *et al.*

Corresponding authors: Gengwen Tan, tangw55@mail.sysu.edu.cn;

Shengfa Ye, shengfa.ye@dicp.ac.cn.

Content

[Experimental section S2](#_Toc122045560)

[Selected molecular structures of the new compounds S10](#_Toc122045561)

[Selected IR and NMR spectra of **3** S14](#_Toc122045562)

[Computation Details S16](#_Toc122045563)

[Crystallographic data and refinement results S23](#_Toc122045564)

[Selected IR and NMR spectra S26](#_Toc122045565)

[Reference S35](#_Toc122045574)

**Experimental section**

General considerations: All experiments were carried out under dry oxygen-free nitrogen using standard Schlenk techniques or in a N_2_ filled-glove box unless otherwise stated. Solvents were dried by standard methods and stored in activated 4 Å molecule sieve in the glovebox. Commercially available reagents were purchased from Energy Chemical and used as received. M^s^Fluid*^t^*^Bu^-Li(thf)_2_ [1], M^s^Fluid^*^-Li(thf)_2_ [2], and Cr(NMe_3_)(CO)_5_ [3] were synthesized according to the reported procedure.

**NMR measurements**

The NMR spectra were recorded on Bruker spectrometers (AV400) referenced to residual solvent signals as internal standards. Chemical shift values for protons are referenced to the residual proton resonance of CDCl_3_ (*δ*: 7.26), C_6_D_6_ (*δ*: 7.16); chemical shift values for carbons are referenced to the carbon resonance of CDCl_3_ (*δ*: 77.16), C_6_D_6_ (δ: 128.06). NMR multiplicities are abbreviated as follows: s = singlet, d = doublet, t = triplet, q = quartet, sept = septet, m = multiplet, br = broad signal. The solutions of samples in the deuterated solvent were sealed off in a NMR tube under vacuum for measurements. The samples were dissolved in deuterated solvents, and were sealed off in J-Young NMR tubes for measurements.

**Single-Crystal X-ray Diffraction Analyses**

For the single crystal X-ray structure analyses the crystals were each mounted on nylon loops in perfluorinated oil and measured in a cold N_2_ flow. The X-ray diffraction data of **1**, **2**, **3**, **4** and **7** was collected on a Bruker D8 VENTURE diffractometer outfitted with a PHOTON-100 CMOS detector using Turbo X-ray Source (TXS) rotating anode MoK*α* radiation (*λ* = 0.71073 Å) at 120 K by chilled nitrogen flow controlled by a KRYOFLEX II low temperature attachment. The X-ray diffraction data of **5**, **6** and **8** was collected on a Bruker D8 VENTURE diffractometer equipped with an Excillum METALJET Ga-K*α* radiation (*λ* = 1.34139 Å) and a PHOTON II CMOS detector with a Helios Multi-layer Optic monochromator at low temperature using an Oxford Cryosystems Cryostream 800 cryostat. The structures were solved by direct methods and all refined on *F*^2^ with the SHELX-2019 software package [4] embedded in Olex 2-1.5 [5]. The positions of the H atoms were calculated and considered isotropically according to a riding model. Seriously disordered solvent molecules in the crystal lattices were omitted with PLATON SQUEEZE [6].

**Elemental Analysis**

Element analyses were performed on an ElementarVario EL III instrument.

**Magnetic Measurements**

Magnetic susceptibility data were measured from powder samples of a solid material of **3** in the temperature range 2−300 K by using a Superconducting Quantum Interference Device (SQUID) with a field of 1 T (MPMS-7, Quantum Design; calibrated with a standard palladium reference sample; error <2%).

**UV-vis-NIR Absortoption Spectra**

UV-vis-NIR measurements were carried out on Shimadzu spectrometer (UV-3600i Plus) with a THF solution of **3** (1.014 mM) at room temperature.

**Synthesis of M^s^Fluid*^t^*^Bu^-BiCl_2_ (1)**

In the glovebox, to the flask containing M^s^Fluid-Li(thf)_2_ (2.67 g, 3.00 mmol) and BiCl_3_ (0.95 g, 3.00 mmol) were added THF (40 mL) at –30 ^o^C. The mixture was allowed to warm up to room temperature and stirred for 12 h to get a yellow suspension. The solvent was removed under vacuum to obtain the product as a light-yellow solid containing LiCl. The product containing LiCl is directly used in the subsequent reaction. Pure product without LiCl could be obtained by collecting the solid through filtering the yellow suspension (Yield: 2.05 g, 2.01 mmol, 67%). Light-yellow crystals suitable for X-ray diffraction analysis were obtained from CDCl_3_/*n*-hexane solution at room temperature. ^1^H NMR (CDCl_3_, 400 MHz, 298 K, ppm): *δ* = 1.27 (s, 36H, C(C*H*_3_)_3_), 1.64 (s, 12H, C(C*H*_3_)_2_), 2.41 (s, 4H, C*H*_2_C(CH_3_)_2_), 7.22-7.23 (m, 4H, fluorene-*H*), 7.31-7.33 (m, 4H, fluorene-*H*), 7.51 (d, 2H, fluorene-*H*), 7.53 (s, 2H, fluorene-*H*), 7.58 (s, 1H, Ar-*H*); ^13^C NMR (CDCl_3_, 100 MHz, 298 K, ppm): *δ* = 31.7 (C(*C*H_3_)_3_), 32.9 (C(*C*H_3_)_2_), 35.1 (*C*(CH_3_)_3_), 44.5 (*C*(CH_3_)_2_), 59.3 (C*C*H_2_C(CH_3_)_2_), 65.4 (*C*CH_2_C(CH_3_)_2_), 119.2, 120.4, 120.5, 120.6, 122.2, 125.6, 125.7, 136.7, 151.3, 154.4, 154.6, 154.8, 155.0, 160.9, 213.0 (Ar-*C*). Elemental analysis for C_56_H_65_BiCl_2_ (%): Cacld: C 66.07, H 6.44; C 65.96, H 6.45.

**Synthesis of M^s^Fluid^*^-BiCl_2_ (2)**

In the glovebox, to the Schlenk flask containing M^s^Fluid^*^-Li(thf)_2_ (2.21 g, 2.00 mmol) and BiCl_3_ (0.63 g, 2.00 mmol) were added THF (20 mL) at –30 ^o^C. The mixture was allowed to warm up to room temperature and stirred for 12 h to give a yellow suspension. The solvent was removed under vacuum to obtain the product as a light-yellow solid containing LiCl. The product containing LiCl is directly used in the subsequent reaction. Pure product without LiCl could be obtained by collecting the solid through filtering the cold yellow suspension (–30 ^o^C) (Yield: 2.10 g, 1.70 mmol, 85%). Light-yellow crystals suitable for X-ray diffraction analysis were obtained from CDCl_3_/*n*-hexane solution at room temperature. ^1^H NMR (CDCl_3_, 400 MHz, 298 K, ppm): *δ* = 1.10 (s, 9H, C(C*H*_3_)_2_), 1.11(s, 3H, C(C*H*_3_)_2_), 1.24 (s, 12H, C(C*H*_3_)_2_), 1.28 (s, 12H, C(C*H*_3_)_2_), 1.34 (s, 12H, C(C*H*_3_)_2_), 1.57-1.63 (m, 28H, C*H*_2_C*H*_2_ (16H) + C(C*H*_3_)_2_ (12H)), 2.34 (s, 4H, C*H*_2_C(CH_3_)_2_), 7.07-7.09 (m, 4H, fluorene-*H*), 7.47 (s, 4H, fluorene-*H*), 7.50 (s, 1H, Ar-*H*); ^13^C{^1^H, ^13^C} NMR (CDCl_3_, 100 MHz, 298 K, ppm): *δ* = 31.97, 32.00, 32.35, 32.38, 32.4, 32.5 (C(*C*H_3_)_2_), 32.8, 34.5, 34.6, 35.17, 35.24 (*C*H_2_*C*H_2_), 44.1, 59.8 (C*C*H_2_C(CH_3_)_2_), 64.5 (*C*CH_2_C(CH_3_)_2_), 118.1, 118.2, 118.8, 123.1, 123.2, 136.8, 144.7, 144.8, 151.9, 152.1, 154.8, 160.3, 219.3 (Ar-*C*). Elemental analysis for C_72_H_89_BiCl_2_ (%): Cacld: C 70.06, H 7.27; C 69.85, H 7.42.

**Synthesis of M^s^Fluid*^t^*^Bu^-Bi (3)**

At –78 ^o^C, THF (40 mL) was transferred to the Schlenk flask containing **1** (LiCl) (2.12 g, 2.00 mmol) and KC_8_ (0.54 g, 2.00 mmol). The reaction mixture was allowed to warm to room temperature and stirred for 12 h. The obtained deep brown suspension was filtered. The volatiles in the brown filtrate were removed under vacuum and the residue was extracted with toluene (50 mL). The obtained brownish yellow solution was filtered and the filtrate was concentrated to ca. 10 mL. After left at 4 ^o^C for 48 h, a yellow crystalline solid of **3** was isolated by removing the mother liquor and dried under vacuum for 2 h. Yield: 0.80 g, 0.84 mmol, 42%. Yellow crystals suitable for X-ray diffraction analysis were obtained from THF/hexane solution at room temperature. ^1^H NMR (THF-D_8_, 400 MHz, 298 K, ppm): *δ* = –1.06 (s, 1H, Ar-*H*), 1.05 (s, 12H, C(C*H*_3_)_2_), 1.69 (s, 36H, C(C*H*_3_)_3_), 1.87 (s, 4H, C*H*_2_C(CH_3_)_2_), 6.73 (d, 4H, ^4^*J*_H,H_ = 1.44 Hz, fluorene-*H*), 7.49 (dd, 4H, ^3^*J*_H,H_ = 7.94 Hz, ^4^*J*_H,H_ = 1.70 Hz, fluorene-*H*), 8.05 (d, 4H, ^3^*J*_H,H_ = 7.94 Hz, fluorene-*H*); ^13^C{^1^H, ^13^C} NMR (THF-D_8_, 100 MHz, 298 K, ppm): *δ* = –203.5, 27.1 (C(*C*H_3_)_2_), 33.8 (C(*C*H_3_)_3_), 36.0, 44.9, 56.4 (*C*H_2_C(CH_3_)_2_), 62.3 (*C*CH_2_C(CH_3_)_2_), 114.7, 124.0, 127.1, 135.8, 151.9, 160.2, 177.0, 177.9, 217.7. Elemental analysis for C_56_H_65_Bi (%): Cacld: C 71.02, H 6.92; C 71.48, H 6.48.

**Synthesis of M^s^Fluid^*^-Bi (4)**

At –78 ^o^C, THF (40 mL) was transferred to the Schlenk flask containing **2** (2.55 g, 2.00 mmol) and KC_8_ (0.54 g, 2.00 mmol). The reaction mixture was allowed to warm to room temperature and stirred for 12 h. The obtained deep brown suspension was filtered. The volatiles in the brown filtrate were removed under vacuum and the residue was extracted with *n*-hexane (50 mL). The obtained brownish yellow solution was filtered and the filtrate was concentrated to ca. 10 mL. After left at 4 ^o^C for 48 h, a yellow crystalline solid of **4** was isolated by removing the mother liquor and dried under vacuum for 2 h. Yield: 1.07 g, 0.92 mmol, 46%. Yellow crystals suitable for X-ray diffraction analysis were obtained from *n*-hexane solution at room temperature. ^1^H NMR (C_6_D_6_, 400 MHz, 298 K, ppm): *δ* = –0.63 (s, 1H, Ar-*H*), 1.12 (s, 12H, C(C*H*_3_)_2_), 1.14 (s, 12H, C(C*H*_3_)_2_), 1.48 (s, 12H, C(C*H*_3_)_2_), 1.59 (s, 12H, C(C*H*_3_)_2_), 1.75-1.99 (m, 20H, CC*H*_2_C(CH_3_)_2_ (4H) + C*H*_2_C*H*_2_ (16H)), 2.13 (s, 12H, C(C*H*_3_)_2_), 7.20 (s, 4H, fluorene-*H*), 8.39 (s, 4H, fluorene-*H*). ^13^C{^1^H, ^13^C} NMR (C_6_D_6_, 100 MHz, 298 K, ppm): *δ* = –189.5, 32.7, 33.6, 33.7, 34.0, 34.6, 35.7, 35.9, 38.3, 44.0, 56.6 (*C*H_2_C(CH_3_)_2_), 60.8 (*C*CH_2_C(CH_3_)_2_), 114.9, 121.3, 140.1, 145.6, 145.8, 157.3, 172.4, 176.1, 228.5. Elemental analysis for C_72_H_89_Bi (%): Cacld: C 74.33, H 7.71; C 74.59, H 7.42.

**Synthesis of M^s^Fluid*^t^*^Bu^-Bi(SPh)_2_ (5)**

In a N_2_-filled glove box, **3** (0.28 g, 0.30 mmol,) and PhSSPh (0.079 g, 0.36 mmol) were placed in a Schlenk flask, and toluene (30 mL) was added at –30 ^o^C. The mixture was stirred at room temperature for 12 h. The yellow solution was filtered and the solvent was removed under vacuum. The residue was extracted with *n*-hexane (40 mL). The yellow extraction was filtered and the filtrate was concentrated to ca. 25 mL and left at –20 ^o^C to afford the crystalline product, which was cropped by removing the mother liquor and dried under vacuum for 2 h. Yield: 0.12 g, 0.10 mmol, 34%. Yellow crystals suitable for X-ray diffraction analysis were obtained from toluene/*n*-hexane solution at room temperature. ^1^H NMR (CDCl_3_, 400 MHz, 298 K, ppm): *δ* = 1.29 (s, 36H, C(C*H*_3_)_3_), 1.64 (s, 12H, C(C*H*_3_)_2_), 2.39 (s, 4H, C*H*_2_C(CH_3_)_2_), 6.00 (dd, 4H, ^3^*J*_H,H_ = 7.91 Hz, ^4^*J*_H,H_ = 1.88 Hz, fluorene-*H*), 6.71-6.79 (m, 6H, fluorene-*H*), 7.09-7.41 (m, 10H, Ph-*H*), 7.50 (s, 1H, Ar-*H*), 7.60 (br, 2H, fluorene-*H*); ^1^H NMR (CDCl_3_, 100 MHz, 298 K, ppm): *δ* = 31.8, 33.0, 35.1, 118.3, 120.0, 122.2, 124.9, 125.4, 127.4, 135.1, 137.4. Much less than expected carbon signals were observed, most probably due to the long relaxation times of these carbon nuclei. Elemental analysis for C_68_H_75_BiS_2_ (%): Cacld: C 70.08, H 6.49; C 70.28, H 6.42.

**Synthesis of M^s^Fluid*^t^*^Bu^-Bi(SePh)_2_ (6)**

In a N_2_-filled glove box, **3** (0.28 g, 0.30 mmol,) and PhSeSePh (0.011 g, 0.36 mmol) were placed in a flask, and toluene (30 mL) was added at –30 ^o^C. The mixture was stirred at room temperature for 12 h. The yellow solution was filtered and the solvent was removed under vacuum. The residue was extracted with *n*-hexane (40 mL). The yellow extraction was filtered and the filtrate was concentrated to ca. 25 mL and left at –20 ^o^C to afford the crystalline product, which was cropped by removing the mother liquor and dried under vacuum for 2 h. Yield: 0.14 g, 0.11 mmol, 37%. Yellow crystals suitable for X-ray diffraction analysis were obtained from toluene/*n*-hexane solution at room temperature. ^1^H NMR (CDCl_3_, 400 MHz, 298 K, ppm): *δ* = 1.33, 1.36 (close two signals, 36H, C(C*H*_3_)_3_), 1.66 (s, 12H, C(CH_3_)_2_), 2.38, 2.39 (close two signals, 4H, C*H*_2_C(CH_3_)_2_), 6.26 (d, 4H, ^3^*J*_H,H_ = 7.06 Hz, fluorene-*H*), 6.73 (t, 4H, ^3^*J*_H,H_ = 7.45 Hz, fluorene-*H*), 6.86 (t, 2H, ^3^*J*_H,H_ = 7.38 Hz, fluorene-*H*), 7.18-7.42 (m, 10H, Ph-*H*), 7.53 (s, 1H, Ar-*H*), 7.65 (d, 4H, ^3^*J*_H,H_ = 7.72 Hz, fluorene-*H*); 31.7, 31.8 (C(*C*H_3_)_3_), 32.9, 33.0 (*C*(CH_3_)_3_), 35.1 (C(*C*H_3_)_2_), 42.4, 44.1 (*C*(CH_3_)_2_), 58.7, 62.2 (*C*H_2_C(CH_3_)_2_), 66.2, 67.7 (*C*CH_2_C(CH_3_)_2_), 118.2, 119.8, 120.5, 121.8, 122.3, 124.9, 125.1, 125.7, 127.6, 129.8, 136.6, 136.7, 138.1, 150.5, 151.0, 151.3, 152.1, 153.9, 155.3, 156.7, 160.7, 181.7. Elemental analysis for C_68_H_75_BiSe_2_ (%): Cacld: C 64.86, H 6.00; C 65.28, H 6.32.

**Synthesis of M^s^Fluid*^t^*^Bu^-Bi→Fe(CO)_4_ (7)**

In the glovebox, M^s^Fluid-Bi (0.38 g, 0.41 mmol) and Fe_2_(CO)_9_ (0.32 g, 0.89 mmol) were dissolved in THF (30 mL) at –30 ^o^C. The mixture was allowed to warm up to room temperature and stirred for 12 h. The formed purple solution was filtered and then the solvent was removed under vacuum. The residue was extracted with toluene (30 mL). The purple solution was filtered and the filtrate was concentrated to 15 mL before placed at –20 ^o^C. The product crystallized as dark-purple solid of **7** after 24 h, which were collected by removing the mother liquor and dried under vacuum. Yield: 0.15 g, 0.13 mmol, 35 %. Purple crystals suitable for X-ray diffraction analysis were obtained from toluene/*n*-hexane solution at room temperature. ^1^H NMR (C_6_D_6_, 400 MHz, 298 K, ppm): *δ* = 1.22 (s, 18H, C(C*H*_3_)_3_), 1.34 (s, 18H, C(C*H*_3_)_3_), 1.51 (s, 6H, C(C*H*_3_)_2_), 1.72 (s, 6H, C(C*H*_3_)_2_), 2.22 (d, 2H, ^2^*J*_H,H_ = 13.85 Hz, C*H*_2_C(CH_3_)_2_), 2.26 (d, 2H, ^2^*J*_H,H_ = 13.85 Hz, C*H*_2_C(CH_3_)_2_), 7.00-7.06 (m, 2H, fluorene-*H*), 7.29-7.35 (t, 4H, ^3^*J*_H,H_ = 7.28 Hz, fluorene-*H*), 7.41 (s, 3H, fluorene-*H* (2H) + Ar-*H* (1H)), 7.46 (d, 2H, ^3^*J*_H,H_ = 7.69 Hz, fluorene-*H*), 7.55 (s, 2H, fluorene-*H*); ^13^C NMR (C_6_D_6_, 100 MHz, 298 K, ppm): *δ* = 31.8, 32.0 (C(*C*H_3_)_3_), 34.4, 34.8, 35.1, 44.2, 56.3, 65.2, 117.1, 120.8, 122.2, 122.5, 122.7, 125.1, 126.8, 136.2, 140.2, 149.9, 151.1, 151.2, 152.7, 153.3, 158.4, 180.7, 197.2, 224.0 (*C*O). Elemental analysis for C_60_H_65_BiFeO_4_ (%): Cacld: C 64.63, H 5.88; C 64.23, H 6.12.

**Synthesis of M^s^Fluid*^t^*^Bu^-Bi→Cr(CO)_5_ (8)**

In the glovebox, M^s^Fluid*^t^*^Bu^-Bi (0.47 g, 0.50 mmol) and Cr(NMe_3_)(CO)_5_ (0.15 g, 0.6 mmol) were dissolved in toluene at –30 ^o^C and stirred for 12 h. The purple solution was filtered and then the solvent was removed under vacuum. The residue was washed with *n*-hexane (30 mL) and the remaining solid was extracted with toluene (40 mL). The purple extraction was filtered and the filtrate was concentrated to ca. 15 mL before placed at –20 ^o^C. The product was obtained as a purple solid after 24 h, which was collected by removing the mother liquor and dried under vacuum for 2 h. Yield: 0.12 g, 0.10 mmol, 20%. Purple crystals suitable for X-ray diffraction analysis were obtained from toluene/*n*-hexane solution at room temperature. In C_6_D_6_ solution, the complex **8** exists as an equilibrium as **8**, **3** and (solvent)Cr(CO)_5_, which makes it difficult to assign the signals correctly. Moreover, in THF-D_8_ solution, complete conversion of **8** to **3** was observed, suggesting the weak coordination between the bismuthinidene and Cr(CO)_5_ fragment. Elemental analysis for C_61_H_65_BiCrO_5_ (%): Cacld: C 64.63, H 5.88; Found: C 64.23, H 5.54.

# Selected molecular structures of the new compounds


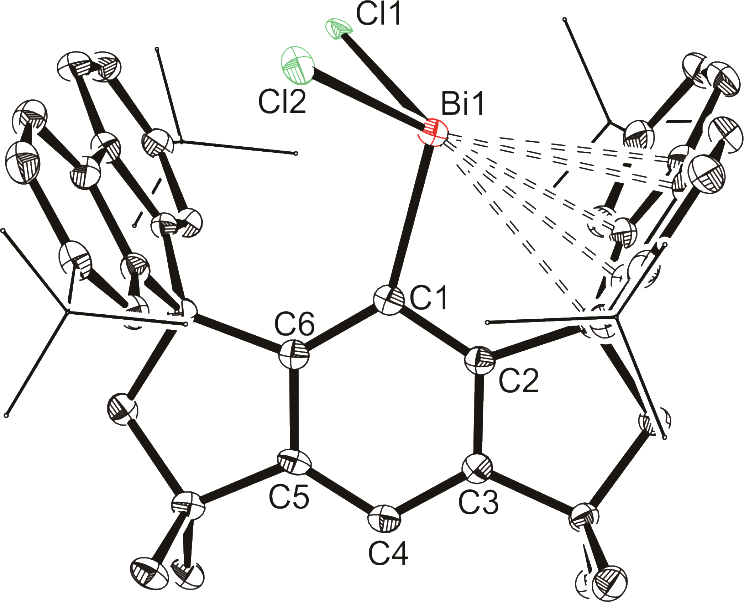


**Figure S1.** Thermal ellipsoid drawing of the molecular structure of M^s^Fluid*^t^*^Bu^-BiCl_2_ (**1**) at 50% probablity. Hydrogen and disorder atoms are omitted for clarity. Selected bond lengths (Å) and angles (^o^): C1–Bi1 2.275(5), Cl1–Bi1 2.528(5), Cl2–Bi1 2.485(6), C1–C2 1.413(8), C2–C3 1.402(8), C3–C4 1.377(8), C4–C5 1.380(8), C5–C6 1.399(7), C1–C6 1.428(8); C2-C1-Bi1 108.5(4), C6-C1-Bi1 134.8(4). The Bi center features an interaction with one of flanking fluorenyl moieties [7].


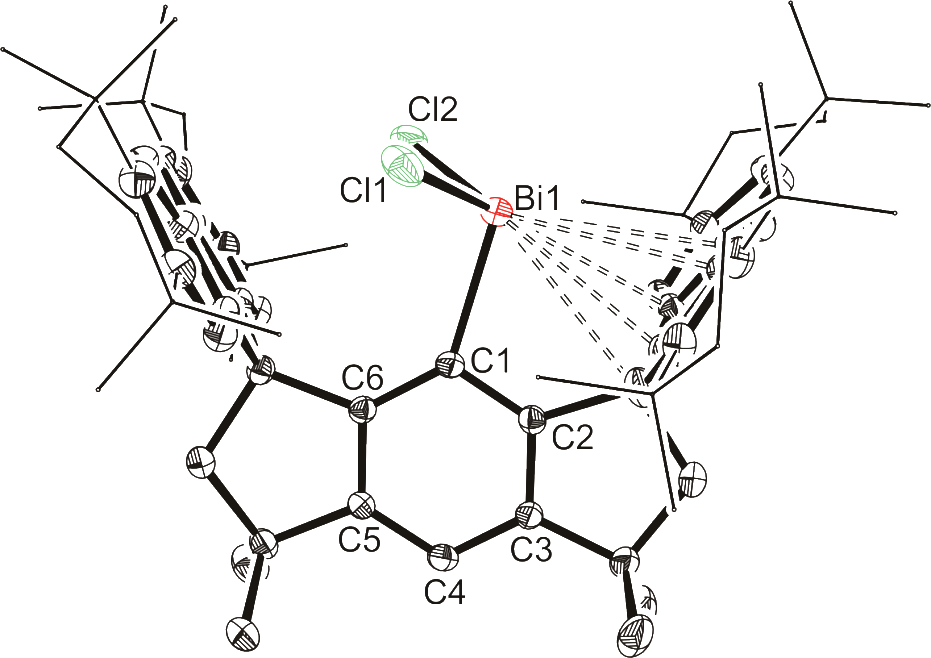


**Figure S2.** Thermal ellipsoid drawing of the molecular structure of M^s^Fluid^*^-BiCl_2_ (**2**) at 50% probablity. Hydrogen and disorder atoms are omitted for clarity. Selected bond lengths (Å) and angles (^o^): C1–Bi1 2.280(4), Cl1–Bi1 2.5032(13), Cl2–Bi1 2.5187(17), C1–C2 1.404(5), C2–C3 1.392(5), C3–C4 1.386(5), C4–C5 1.384(5), C5–C6 1.396(5), C1–C6 1.410(5); C2-C1-Bi1 107.4(2), C6-C1-Bi1 134.7(3). The Bi center features an interaction with one of flanking fluorenyl moieties [7].

**Figure S3.** Depiction of two neighboring molecules of **3** exhibiting a face-to-face interaction.


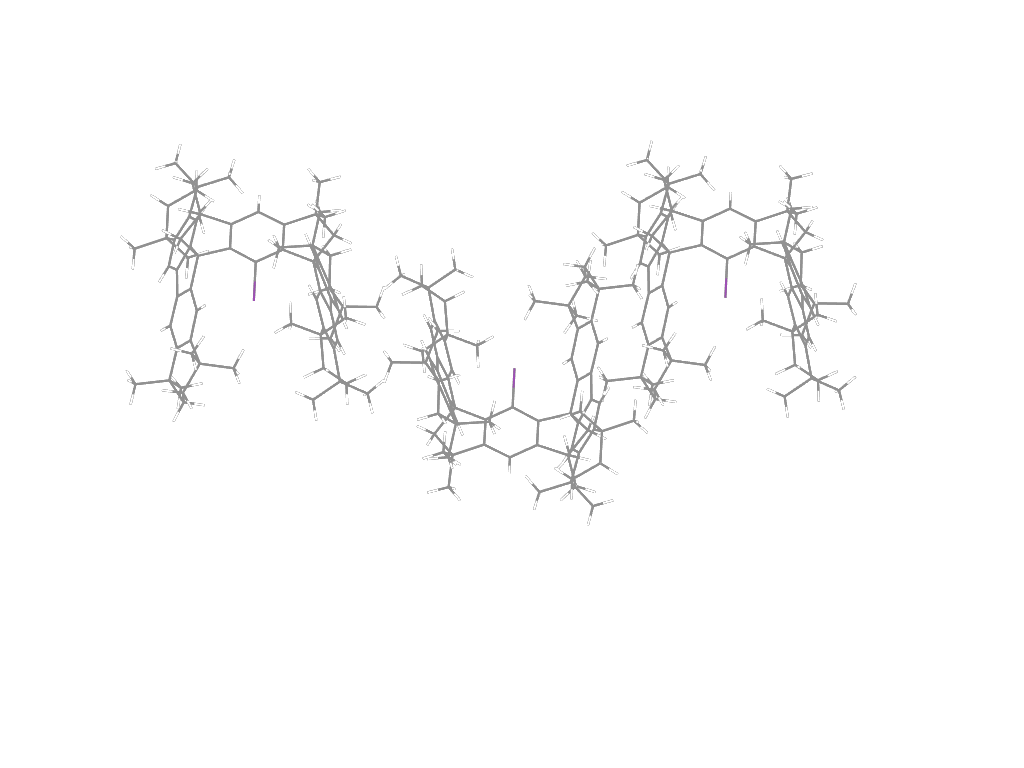


**Figure S4.** Depiction of the three neighboring molecules of **4**.


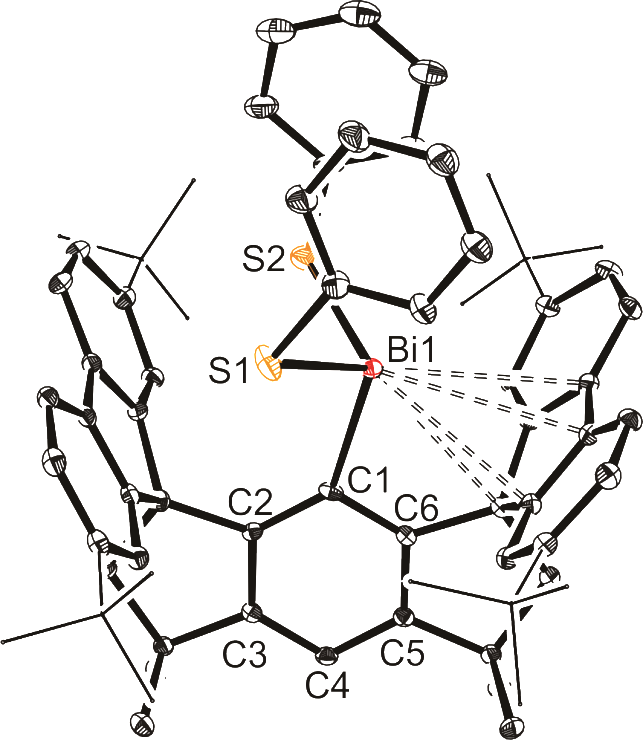


**Figure S5.** Thermal ellipsoid drawing of the molecular structure of M^s^Fluid*^t^*^Bu^-Bi(SPh)_2_ (**5**) at 50% probablity. Hydrogen and disorder atoms are omitted for clarity. Selected bond lengths (Å) and angles (^o^): C1–Bi1 2.297(3), S1–Bi1 2.5376(14), S2–Bi1 2.5365(14), C1–C2 1.407(5), C2–C3 1.402(5), C3–C4 1.387(5), C4–C5 1.385(5), C5–C6 1.399(5), C1–C6 1.398(5); C1-Bi1-S1 104.20(9), C1-Bi1-S2 99.13(9), C2-C1-Bi1 134.7(2), C6-C1-Bi1 107.4(2). The Bi center features an interaction with one of flanking fluorenyl moieties [7].


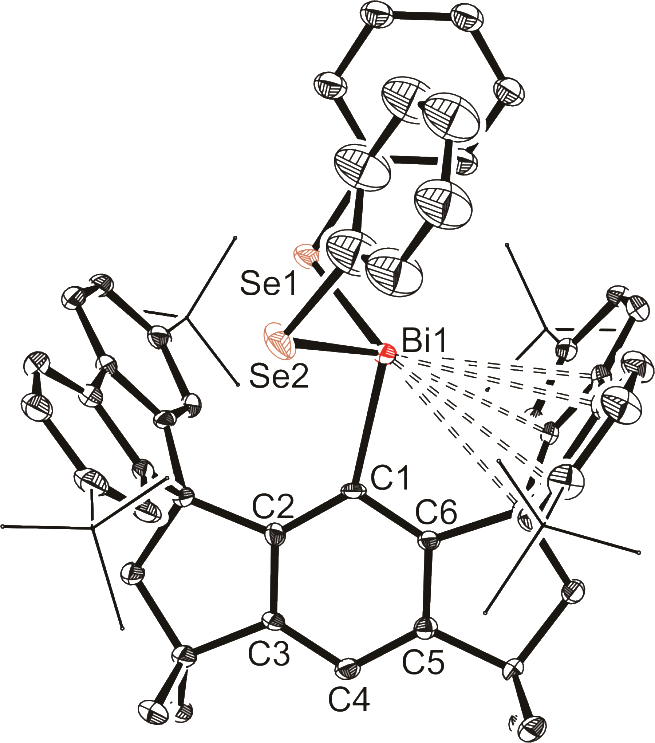


**Figure S6.** Thermal ellipsoid drawing of the molecular structure of M^s^Fluid*^t^*^Bu^-Bi(SePh)_2_ (**6**) at 50% probablity. Hydrogen and disorder atoms are omitted for clarity. Selected bond lengths (Å) and angles (^o^): C1–Bi1 2.294(5), Se1–Bi1 2.6617(8), Se2–Bi1 2.6738(9), C1–C2 1.421(7), C2–C3 1.373(7), C3–C4 1.380(7), C4–C5 1.386(7), C5–C6 1.387(7), C1–C6 1.412(7); C1-Bi1-Se1 103.33(12), C1-Bi1-Se2 100.84(13), C2-C1-Bi1 132.5(4), C6-C1-Bi1 110.8(3). The Bi center features an interaction with one of flanking fluorenyl moieties [7].


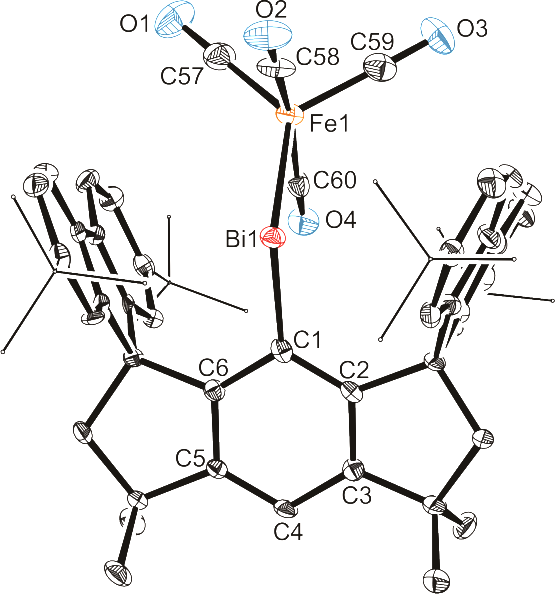


**Figure S7.** Thermal ellipsoid drawing of the molecular structure of M^s^Fluid*^t^*^Bu^-Bi→Fe(CO)_4_ (**7**) at 50% probablity. Hydrogen and disorder atoms are omitted for clarity. Selected bond lengths (Å) and angles (^o^): C1–Bi1 2.286(6), Bi1–Fe1 2.5501(9), Fe1-C57 1.789(8), Fe1–C59 1.795(8), Fe1–C58 1.805(7), Fe1–C60 1.815(7), C1–C2 1.392(9), C2–C3 1.389(9), C3–C4 1.395(9), C4–C5 1.379(9), C5–C6 1.402(8), C1–C6 1.401(8); C1-Bi1-Fe1 113.86(15), C2-C1-Bi1 120.2(4), C6-C1-Bi1 120.0(4).


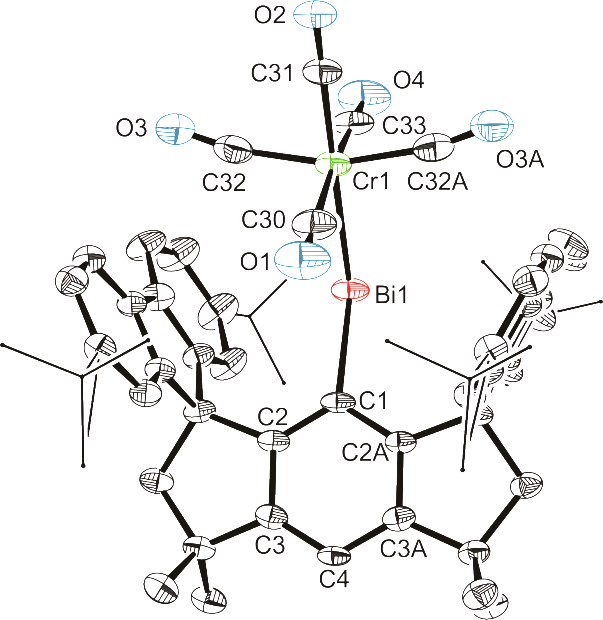


**Figure S8.** Thermal ellipsoid drawing of the molecular structure of M^s^Fluid*^t^*^Bu^-Bi→Cr(CO)_5_ (**8**) at 50% probablity. Hydrogen and disorder atoms are omitted for clarity. Selected bond lengths (Å) and angles (^o^): C1–Bi1 2.317(6), Bi1–Cr1 2.7263(14), Cr(1)-C(31) 1.859(9), Cr(1)-C(32) 1.889(7), Cr(1)-C(33) 1.892(10), Cr(1)-C(30) 1.929(10), C1–C2 1.397(6), C2–C3 1.392(6), C3–C4 1.385(6), C4–C3A 1.385(6); C1-Bi1-Cr1 120.56(16), C2-C1-Bi1 119.4(3), C2A-C1-Bi1 119.4(3). Symmetry transformations used to generate equivalent atoms labeled with ‘A’: x, -y+1/2, z.

# Selected IR and NMR spectra of 3

**Figure S9.** HSQC spectrum of **3** in THF-D_8_ at 298 K, showing the correlation at the Ar-*CH* group.


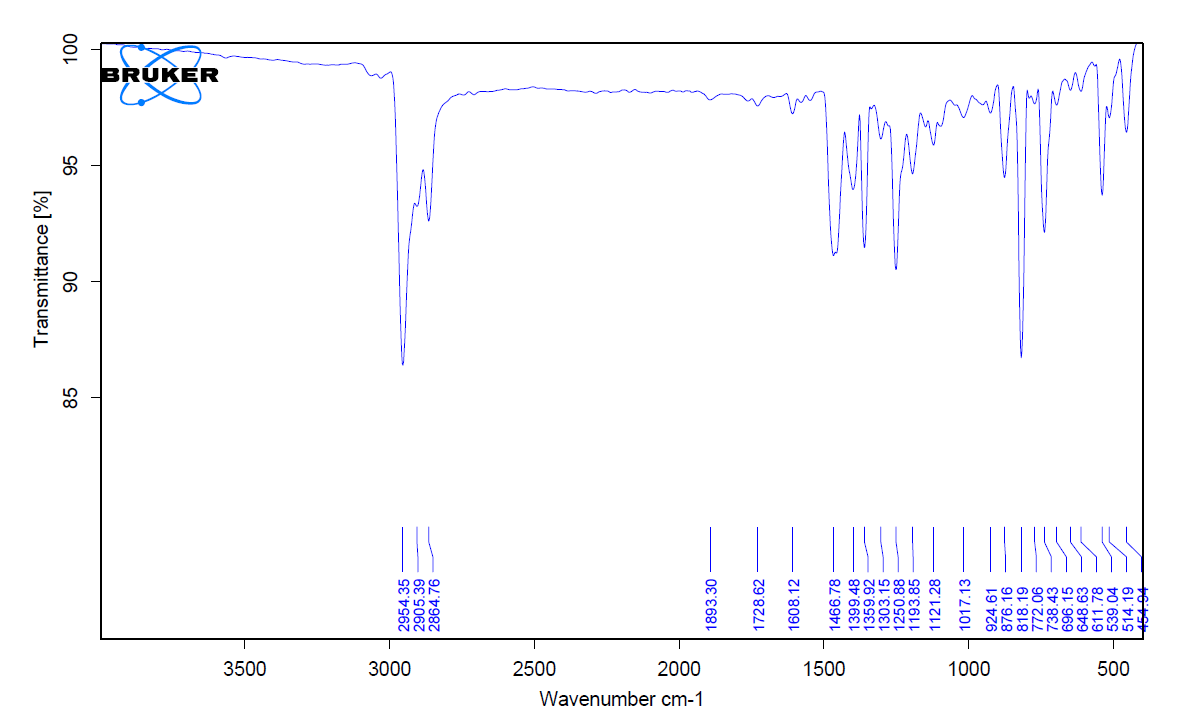


**Figure S10.** Infra-red absorption spectrum of **3** recorded in solid state at room temperature.

**Figure S11.** UV-vis-NIR Absortoption Spectra of 1.014 mM of **3** in THF at 298K.

# Computation Details

All calculations were performed by using the ORCA quantum chemical program package [8]. To properly account for relativistic effects of Bi, the scalar relativistic second-order Douglas-Kroll-Hess (DKH) Hamiltonian [9, 10] in combination with all-electron sarc-dkh-tzvp relativistic basis set for Bi and dkh-def2-svp basis set [11] for other atoms were employed. DFT calculations and noncovalent interaction (NCI) analyses were undertaken by using the PBE density functional. The intramolecular noncovalent interactions were depicted by plots of the reduced density gradient verses the sign of the second eigenvalue of the hessian matrix(sign(λ_2_)) multiplied by the electron density(*ρ*) (Figure S12) [12-14]. Complete active space self-consistent field (CASSCF) [15-17]/*N*-electron valence perturbation theory up to the 2nd order (NEVPT2) [18] computations were also employed the DKH Hamiltonian and the same basis sets. The active space was chosen to distribute 12 electrons into 11 orbitals including Bi-C σ and σ*, Bi 6s and 6p orbitals as well as the six π orbitals of the phenyl ring.


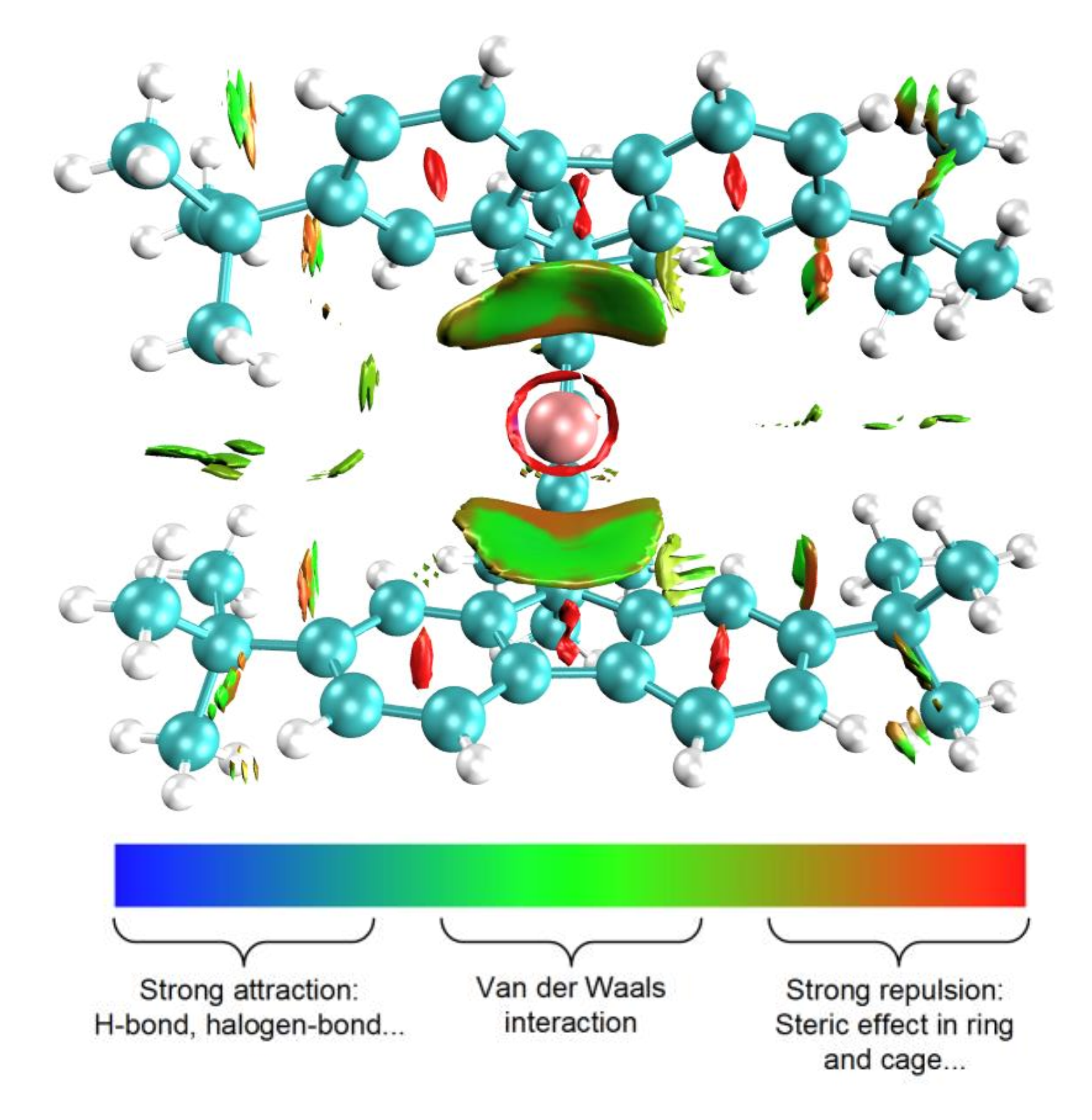


**Figure S12.** The reduced gradient isosurface (*s*(**r**) = 0.5) is colored on a blue-green-red scale in accordance with the value of sign(λ_2_)*ρ*, ranging from -0.04 to 0.02 au.

Noncovalent interaction analyses (Figure S12) show that there are notable van der Waals interactions between the Bi center and the flanking fluorenyl moieties. To deeply explore the role of dispersion interaction in stabilizing the Bi center, we carried out geometry optimizations of complex **3** with and without D3BJ corrections proposed by Grimme and Schreiner[19]. As shown in Figure S13, compared to the computed geometry with D3BJ corrections, calculations without D3BJ corrections predicted much longer distances of the Bi center to the carbon atoms of the two flanking fluorenyl groups. In addition, D3BJ corrections give strong noncovalent interactions of −129 kcal/mol. Therefore, dispersion interactions between the two flanking fluorenyl groups and the Bi center are essentially attractive in nature, which make the Bi center more isolated and increase its stability.


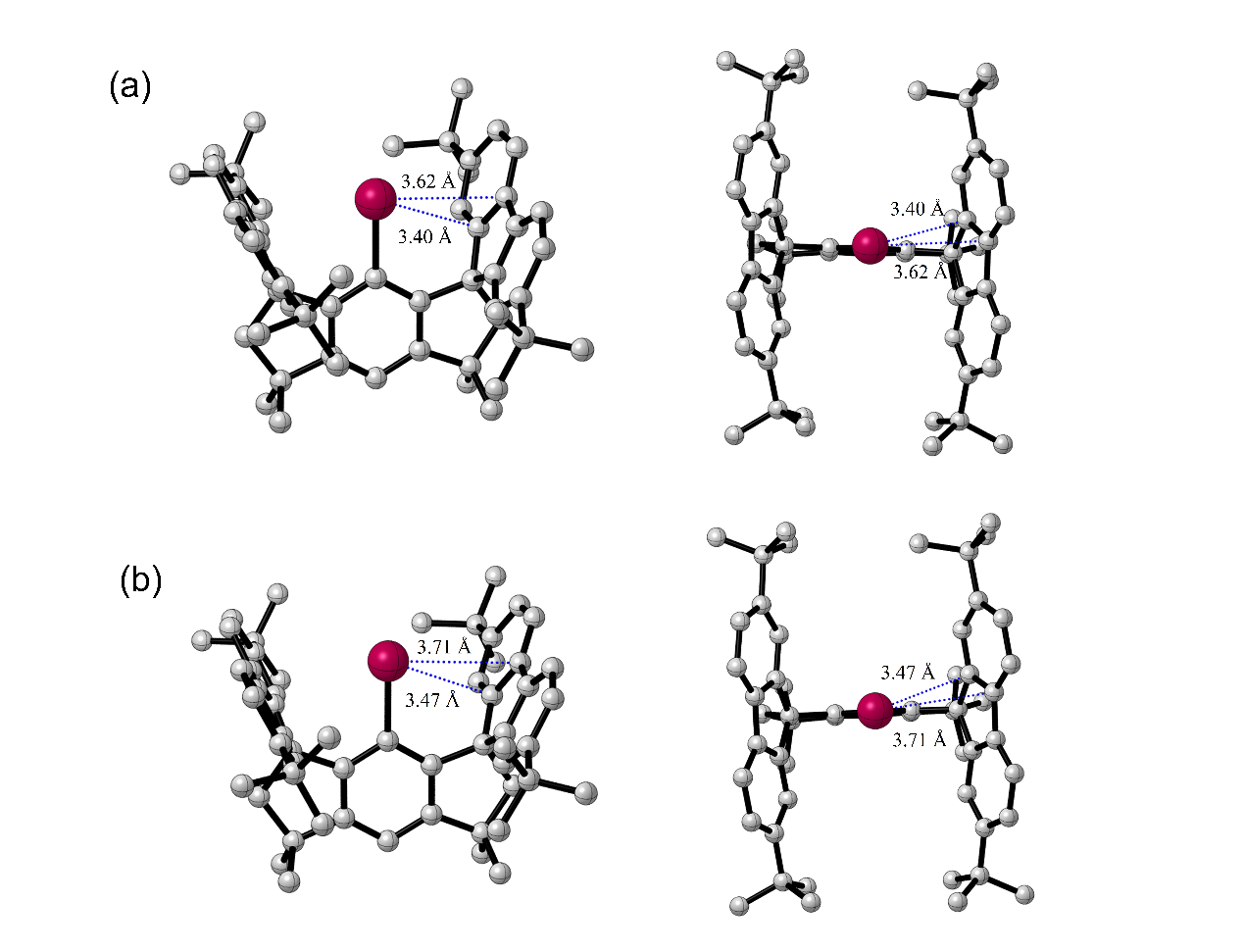


**Figure S13.** (a) the optimized geometry with D3BJ corrections (b) the optimized geometry without D3BJ corrections

CASSCF(12,11)/NEVPT2 computations showed that **3ʹ** possesses a triplet ground state, and the first triplet excited state arising from the excitations of Ph π_2,3_ → Bi 6p_x,y_ lies 86.8 kcal/mol higher in energy. The first and second singlet states are open- and closed-shell singlet states that are 18.4 and 18.5 kcal/mol above the triplet ground state, respectively. Furthermore, **3ʹ** was predicted to feature an exceeding large, positive axial zero-field splitting of more than 7800 cm^–1^ and a vanishing *E/D* (Table S1). Specifically, ab inito calculations reveals that intra-SOMO (SOMO = singly occupied molecular orbital) spin-flip transitions make dominant contributions to *D* (Table S2). Based on the prediected *D* and *E/D*, we simulated varable-temparature magnetic susceptibility data. As can be seen in Figure S14, the χT value at room temperature is less 0.1 emu•K, which is by far below the expected value of 1 emu•K typically observed for triplet systems. More crtically, the temperature that makes ꭓT saturated is as high as 30000 K. Consequently, the marginal value at room temperature, whose magnetitude is close to typical TIP terms cannot be accurately measured by using SQUID.

**Table S1.** *D* and *E*/*D* values of **3ʹ** by CASSCF/NEVPT2. Calculation with Different Conditions

| Multiplicity | Roots | *D*/ cm^-1^ | *E*/*D* |
| --- | --- | --- | --- |
| 1, 3 | 3, 3 | 9370 | 0.04 |
| 1, 3 | 6, 6 | 8430 | 0.06 |
| 1, 3 | 10, 10 | 7810 | 0.001 |

**Table S2.** Breakdown of Main Contributions to *D* Value from Excited States of Compound **3ʹ** obtained by CASSCF/NEVPT2. Calculation averaging ten singlets and ten triplets.

| Excitation | Excited states | Excitation energy/cm^-1^ | Contribution to D-tensor/cm^-1^ |
| --- | --- | --- | --- |
| $p_{x}\to\bar{p_{y}}$and $p_{y}\to\bar{p_{x}}$ | ${{}^{1}A}_{1}\left( \text{Ⅰ} \right)$ | 6080 | 2278 |
| $p_{x}\to\bar{p_{x}}$ and $p_{y}\to\bar{p_{y}}$ | ${{}^{1}A}_{2}$ | 6350 | 59 |
| $p_{x}\to\bar{p_{y}}$and $p_{y}\to\bar{p_{x}}$ | ${{}^{1}A}_{1}\left( \text{Ⅱ} \right)$ | 13490 | 8561 |
| $\sigma_{z}\to p_{y}$ | ${{}^{3}B}_{2}$ | 30480 | 336 |
| $\sigma_{z}\to p_{x}$ | ${{}^{3}B}_{1}$ | 32620 | 349 |
| $\sigma_{z}\to\bar{p_{y}}$ | ${{}^{1}B}_{2}$ | 34170 | -351 |
| $\sigma_{z}\to\bar{p_{x}}$ | ${{}^{1}B}_{1}$ | 36040 | -357 |

Here, a real p-orbital without and with an overbar indicates that this orbital is occupied by an ɑ and a β electron, respectively.


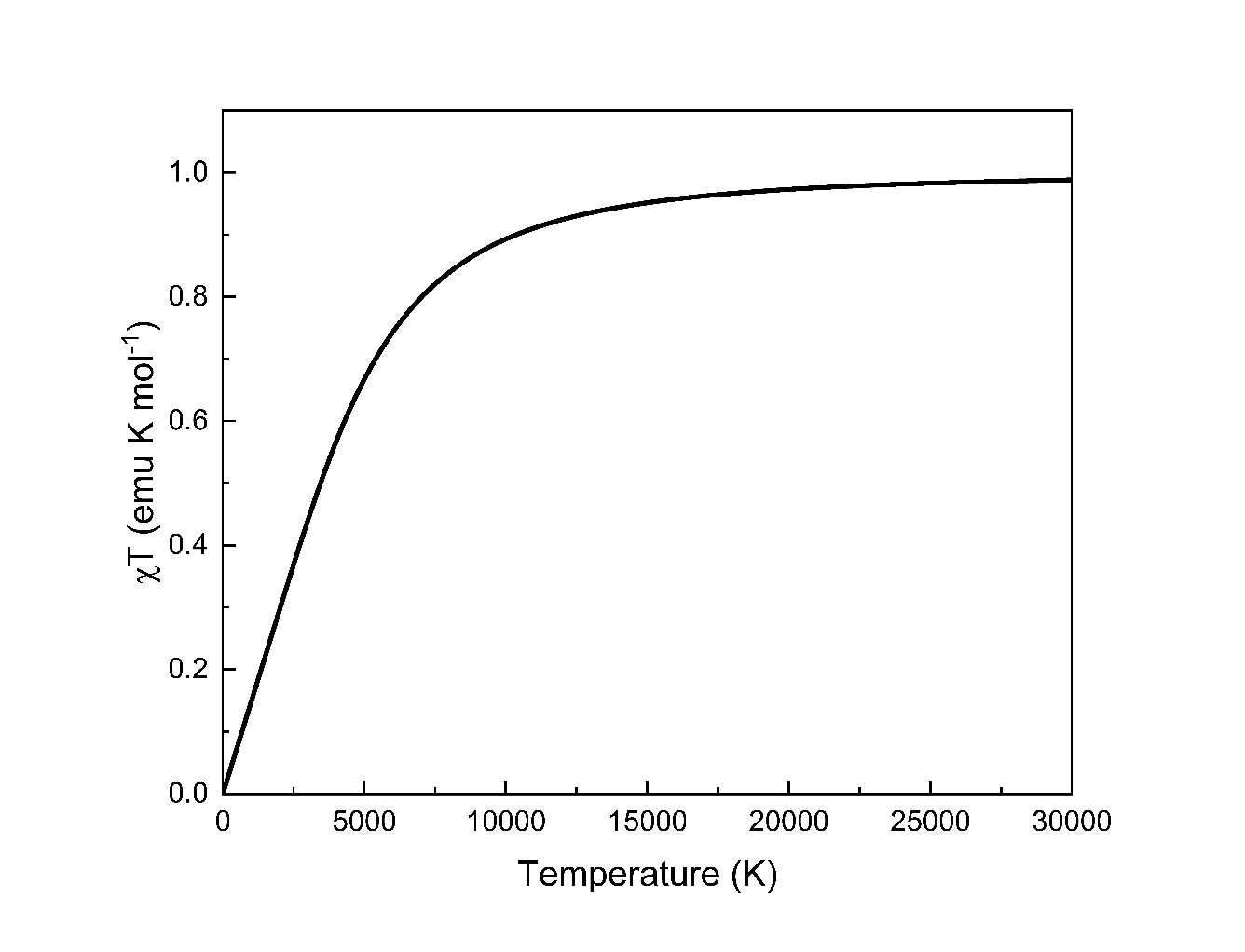


**Figure S14.** Simulated varable-temperature magnetic suscepbility data for *D* = 9400 cm^–1^, *E/D* = 0, *g*_iso_ = 2.0.

ZFSs are composed of two contributions: (1) direct spin-spin coupling (SSC) between a pair of unpaired electrons represents the first order term in the perturbation theory and (2) the second order term originates from spin-orbit coupling (SOC) interaction. In the present case, the SSC interaction can be ignored and the very large ZFS results form strong SOC interactions between the ground state and the excited states, because the effective SOC constant of Bi reaches as high as 12000 cm^-1^ [20][21].

To probe the origin of the large ZFS, we carried out ligand field analyses for which the ground state ^3^A_2_ and three intra-SOMO spin-flip excited states ${{}^{1}A}_{2}$, ${{}^{1}A}_{1}\left( \text{Ⅰ} \right)$ and ${{}^{1}A}_{1}\left( \text{Ⅱ} \right)$ were taken into account. Here, the irreducible representations of the C_2v_ point group are employed to denote the ground and excited states. Their many-electron wavefunctions in the basis of normalized slater determinants are given by

$$\left| \left. {{}^{3}A}_{2}, +1 \right\rangle\right.=\left| p_{x}p_{y} \right|$$

$$\left| \left. {{}^{3}A}_{2}, -1 \right\rangle\right.=\left| \bar{p}_{x}\bar{p}_{y} \right|$$

$$\left| \left. {{}^{3}A}_{2}, 0 \right\rangle\right.=\frac{1}{\sqrt{2}}\left( \left| \bar{p}_{x}p_{y} \right|+\left| p_{x}\bar{p}_{y} \right| \right)$$

$$\left| \left. {{}^{1}A}_{2}, 0 \right\rangle\right.=\frac{1}{\sqrt{2}}\left( \left| \bar{p}_{x}p_{y} \right|-\left| p_{x}\bar{p}_{y} \right| \right)$$

$$\left| \left. {{}^{1}A}_{1}\left( \text{Ⅰ} \right), 0 \right\rangle\right.=\cos\theta\left| p_{x}\bar{p}_{x} \right|-\sin\theta\left| p_{y}\bar{p}_{y} \right|$$

$$\left| \left. {{}^{1}A}_{1}\left( \text{Ⅱ} \right), 0 \right\rangle\right.=\sin\theta\left| p_{x}\bar{p}_{x} \right|+\cos\theta\left| p_{y}\bar{p}_{y} \right|$$

Because both ${{}^{1}A}_{1}\left( \text{Ⅰ} \right)$ and ${{}^{1}A}_{1}\left( \text{Ⅱ} \right)$ are linear combinations of $\left| p_{x}\bar{p}_{x} \right|$ and $\left| p_{y}\bar{p}_{y} \right|$, a mixing angle $\text{θ}$ was introduced to depict the relative weight of $\left| p_{x}\bar{p}_{x} \right|$ and $\left| p_{y}\bar{p}_{y} \right|$ in the ${{}^{1}A}_{1}\left( \text{Ⅰ} \right)$ and ${{}^{1}A}_{1}\left( \text{Ⅱ} \right)$ wavefunctions. The SOC Hamiltonian in terms of single electron operator is given by

$$H_{\mathrm{SOC}} = \text{ζ}\sum_{i} \sum_{k=x,y,z} \vec{l}_{k}\left( i \right)\cdot\vec{s}_{k}\left( i \right)$$

Here$,\text{ ζ}$ ~ 12000 cm^-1^is the SOC constant of Bi and$\text{θ}$ is equivalent to π/3. The contribution from each excited state to *D* is calculated by using the second-order perturbation theory.

$$D_{{{}^{1}A}_{1}\left( \text{Ⅱ} \right)}=\Delta E_{\pm}-\Delta E_{0}=-\frac{\left| \left\langle{{}^{3}A}_{2}, \pm1 | H_{SOC} | {{}^{1}A}_{1}\left( \text{Ⅱ} \right), 0 \right\rangle\right|^{2}}{\Delta E\left( {{}^{3}A}_{2}\underset{\to}{}{{}^{1}A}_{1}\left( \text{Ⅱ} \right) \right)}+\frac{\left| \left\langle{{}^{3}A}_{2}, 0 | H_{SOC} | {{}^{1}A}_{1}\left( \text{Ⅱ} \right), 0 \right\rangle\right|^{2}}{\Delta E\left( {{}^{3}A}_{2}\underset{\to}{}{{}^{1}A}_{1}\left( \text{Ⅱ} \right) \right)}=\frac{\left( \sin\theta+\cos\theta\right)^{2}\text{ζ}^{2}}{2\times\Delta E\left( {{}^{3}A}_{2}\underset{\to}{}{{}^{1}A}_{1}\left( \text{Ⅱ} \right) \right)}\approx10220 \mathrm{cm}^{-1}$$

$$D_{{{}^{1}A}_{1}\left( \text{Ⅰ} \right)}=-\frac{\left| \left\langle{{}^{3}A}_{2}, \pm1 | H_{SOC} | {{}^{1}A}_{1}\left( \text{Ⅰ} \right), 0 \right\rangle\right|^{2}}{\Delta E\left( {{}^{3}A}_{2}\underset{\to}{}{{}^{1}A}_{1}\left( \text{Ⅰ} \right) \right)}+\frac{\left| \left\langle{{}^{3}A}_{2}, 0 | H_{SOC} | {{}^{1}A}_{1}\left( \text{Ⅰ} \right), 0 \right\rangle\right|^{2}}{\Delta E\left( {{}^{3}A}_{2}\underset{\to}{}{{}^{1}A}_{1}\left( \text{Ⅰ} \right) \right)}=\frac{\left( \sin\theta-\cos\theta\right)^{2}\text{ζ}^{2}}{2\times\Delta E\left( {{}^{3}A}_{2}\underset{\to}{}{{}^{1}A}_{1}\left( \text{Ⅰ} \right) \right)}\approx1620 \mathrm{cm}^{-1}$$

$$D_{{{}^{1}A}_{2}}=-\frac{\left| \left\langle{{}^{3}A}_{2}, \pm1 | H_{SOC} | {{}^{1}A}_{2}, 0 \right\rangle\right|^{2}}{\Delta E\left( {{}^{3}A}_{2}\underset{\to}{}{{}^{1}A}_{2} \right)}+\frac{\left| \left\langle{{}^{3}A}_{2}, 0 | H_{SOC} | {{}^{1}A}_{2}, 0 \right\rangle\right|^{2}}{\Delta E\left( {{}^{3}A}_{2}\underset{\to}{}{{}^{1}A}_{2} \right)}=\text{0}$$

The dominant contributions to ZFS computed by the second-order perturbation theory originate from the excited singlet ${{}^{1}A}_{1}\left( \text{Ⅰ} \right)$ and ${{}^{1}A}_{1}\left( \text{Ⅱ} \right)$, and the final *D* value is comparable to that predicted by CASSCF(11, 12)/NEVPT2 calcalutions by averaging three singlets and three triplets.

The computed electronic structures of the singlet ground states of **7** and **8** are shown in Figures S15 and S16, respectively. As can be seen from the different percentages of Bi and Fe/Cr of the vacent molecular orbital labeled as Bi 6p_x_, the π-backdonation in **7** is considerably stronger than that in **8**, and the Bi-Fe interaction in **7** possesses more double bond character. In line with this notion, the computed Mayer Bi-Fe bond order of 1.10 appreciablly excceds the Bi-Cr bond order of 0.80, which accounts for the varying Bi-Fe and Bi-Cr bond lengths and bonding strengths.

**
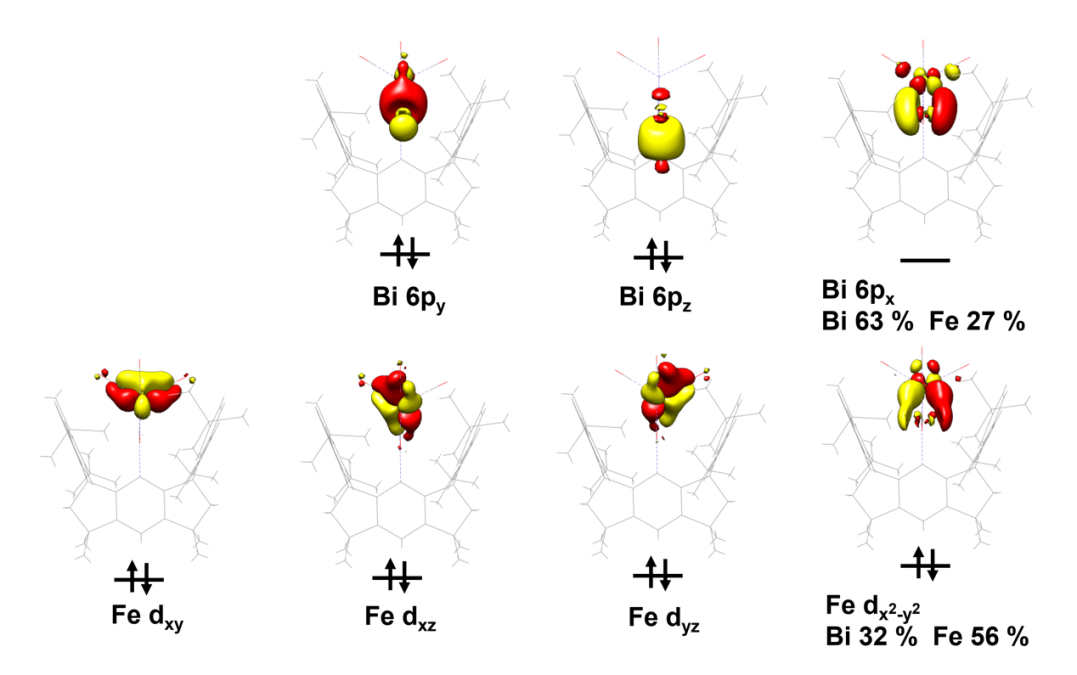
**

**Figure S15.** The molecular orbital diagram of **7**.


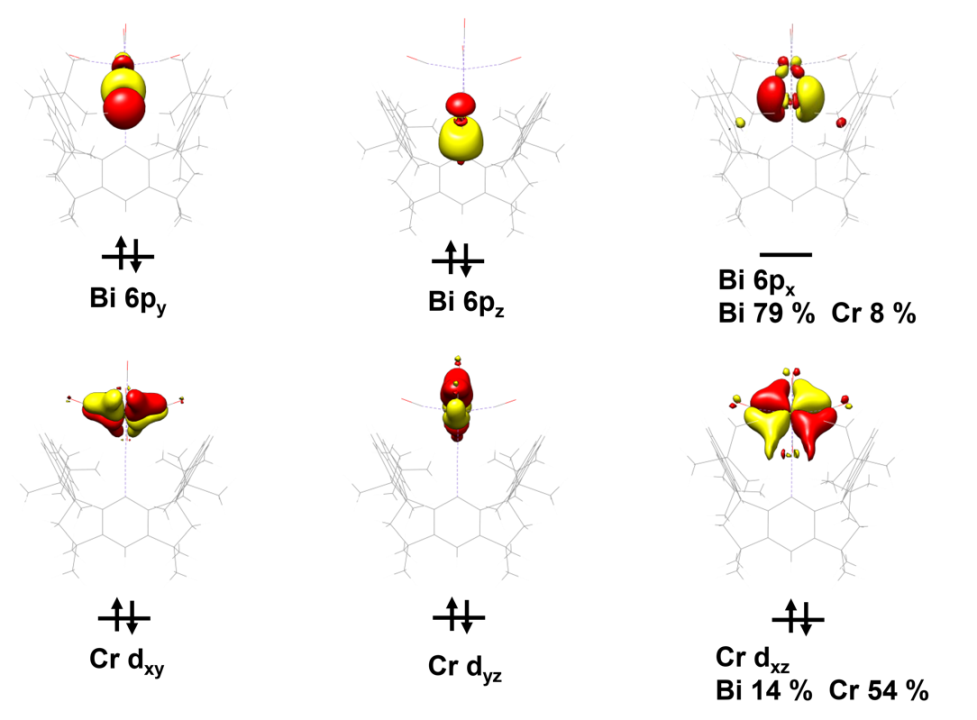


**Figure S16.** The molecular orbital diagram of **8**.

**Simplified model 3’**

Bi 0.000985 0.001375 2.280348

C 0.000933 0.001102 -0.002845

C 1.190428 0.001755 -0.736036

C 1.187204 0.022245 -2.129994

C -0.015973 0.068762 -2.840271

H -0.011140 0.091070 -3.938845

C -1.199366 0.074217 -2.138513

C -1.201866 0.037350 -0.734773

C 3.503361 -0.072547 -1.487653

H 4.272409 0.717848 -1.491255

H 4.043362 -1.034546 -1.474672

C 2.571013 0.012007 -2.729561

C -3.486371 -0.172252 -1.473053

H -3.782960 -1.235416 -1.490282

H -4.415188 0.422000 -1.444096

C -2.594942 0.130735 -2.720173

C -2.611340 0.069221 -0.189343

C -2.959851 -0.980681 0.868032

C -2.690307 -2.348271 0.836235

H -2.112506 -2.772453 0.003577

C -3.133999 -3.178214 1.896162

C -3.875943 -2.577218 2.921033

H -4.242859 -3.209473 3.741445

C -4.142006 -1.225701 2.959891

H -4.705997 -0.792517 3.795259

C -3.662219 -0.417919 1.922307

C -3.670912 1.039635 1.734628

C -4.134530 2.073145 2.555567

H -4.660366 1.844726 3.491678

C -3.915025 3.374556 2.183661

H -4.275810 4.186457 2.829165

C -3.210459 3.705101 1.002975

C -2.761680 2.660017 0.201387

H -2.197204 2.876785 -0.716967

C -2.991440 1.338344 0.556882

C 2.619540 0.036214 -0.192387

C 2.957585 -1.041938 0.814929

C 2.845780 -2.431222 0.653967

H 2.441649 -2.840290 -0.282070

C 3.230077 -3.281842 1.694659

C 3.715844 -2.697523 2.902102

H 4.003914 -3.359658 3.728845

C 3.835941 -1.334308 3.051158

H 4.217815 -0.910276 3.988709

C 3.467635 -0.496541 1.991503

C 3.462142 0.964302 1.855236

C 3.858681 1.981419 2.732883

H 4.298059 1.744307 3.709714

C 3.672186 3.294658 2.345951

H 3.975732 4.093638 3.036431

C 3.089839 3.646051 1.132204

C 2.729266 2.609495 0.250400

H 2.271064 2.845380 -0.720302

C 2.922962 1.295575 0.615272

H -2.802940 1.136286 -3.136118

H -2.750928 -0.598250 -3.535460

H 2.714832 -0.842906 -3.416600

H 2.753162 0.928319 -3.323787

H -3.024094 4.750322 0.733317

H 2.932951 4.695978 0.861766

H 3.146059 -4.370132 1.599700

H -2.915927 -4.251394 1.904047

# Crystallographic data and refinement results

**Table S3.** Crystal data and refinement of **1**-**3**

|  | **1** | **2** | **3** |
| --- | --- | --- | --- |
| CCDC No. | 2227156 | 2227157 | 2227158 |
| formula | C_56_H_65_Cl_2_Bi | C_78_H_103_BiCl_2_ | C_56_H_65_Bi |
| formula weight | 1017.96 | 1320.48 | 947.06 |
| crystal system | Monoclinic | Monoclinic | triclinic |
| space group | *P*2_1_/*c* | *P*2_1_/*c* | *P*-1 |
| *a*/Å | 12.1899(3) | 12.590(2) | 12.6985(7) |
| *b*/Å | 21.6056(7) | 26.557(5) | 14.3454(9) |
| c/Å | 18.3281(6) | 21.128(4) | 15.3412(10) |
| *α*/deg |  |  | 108.610(2) |
| *β*/deg | 91.6780(10) | 99.303(7) | 102.979(2) |
| *γ*/deg |  |  | 106.005(2) |
| *V*/Å^3^ | 4825.0(3) | 6972(2) | 2391.3(3) |
| *Z* | 4 | 4 | 2 |
| *ρ*_calcd_/g∙cm^-3^ | 1.401 | 1.258 | 1.315 |
| *μ*/mm^-1^ | 3.800 | 2.646 | 3.721 |
| *F*(000) | 2072 | 2752 | 968 |
| crystal size/mm^3^ | 0.12 x 0.10x 0.9 | 0.26 x 0.22 x 0.20 | 0.20 x 0.16 x 0.12 |
| *θ* range/deg | 1.919–25.000 | 2.245–27.536 | 2.385-27.504 |
| collected data | 44369 | 76041 | 59633 |
| unique data | 8493 (*R*_int_ = 0.0599) | 16036 (*R*_int_ = 0.0585) | 10979  (*R*_int_ = 0.0548) |
| GOF on *F*^2^ | 1.030 | 0.848 | 1.047 |
| final *R* indices [*I*>2*σ*(*I*)] | *R*_1_ = 0.0443  *wR*_2_ = 0.1103 | *R*_1_ = 0.0476  *wR*_2_ = 0.1380 | *R*_1_ = 0.0461  *wR*_2_ = 0.1192 |
| *R* indices (all data) | *R*_1_ = 0.0582  *wR*_2_ = 0.1203 | *R*_1_ = 0.0691  *wR*_2_ = 0.1577 | *R*_1_ = 0.0428  *wR*_2_ = 0.1261 |
| Largest diff peak/hole (e⋅Å^-3^) | 1.50/–0.95 | 1.57/–0.86 | 1.70/–1.82 |

**Table S4**. Crystal data and refinement of **4**-**6**

|  | **4** | | **5** | | **6** | |
| --- | --- | --- | --- | --- | --- | --- |
| CCDC No. | 2227159 | 2227160 | | 2227161 | |  |
| ormula | C_72_H_89_Bi | | C_68_H_75_BiS_2_ | | C_68_H_75_BiSe_2_ | |
| formula weight | 1163.41 | | 1165.38 | | 1259.18 | |
| crystal system | Triclinic | | Triclinic | | Triclinic | |
| space group | *P*-1 | | *P*-1 | | *P*-1 | |
| *a*/Å | 13.8912(12) | | 12.7409(7) | | 12.2519(8) | |
| *b*/Å | 15.1149(14) | | 15.1725(9) | | 12.8071(8) | |
| c/Å | 16.1711(13) | | 15.7493(9) | | 19.6748(13) | |
| *α*/deg | 87.102(3) | | 90.457(2) | | 99.024(3) | |
| *β*/deg | 89.567(3) | | 93.777(2) | | 98.276(3) | |
| *γ*/deg | 77.306(3) | | 109.133(2) | | 97.148(3) | |
| *V*/Å^3^ | 3308.1(5) | | 2868.7(3) | | 2982.9(3) | |
| *Z* | 2 | | 2 | | 2 | |
| *ρ*_calcd_/g∙cm^-3^ | 1.168 | | 1.349 | | 1.402 | |
| *μ*/mm^-1^ | 2.701 | | 4.655 | | 4.970 | |
| *F*(000) | 1208 | | 1196 | | 1268 | |
| crystal size/mm^3^ | 0.20 x 0.15 x 0.13 | | 0.12 x 0.10 x 0.08 | | 0.16 x 0.12 x 0.10 | |
| *θ* range/deg | 1.918–27.655 | | 2.683–53.954 | | 3.208-54.082 | |
| collected data | 88521 | | 43787 | | 48415 | |
| unique data | 15325 | | 10438  (*R*_int_ = 0.0634) | | 10898  (*R*_int_ = 0.0761) | |
| GOF on *F*^2^ | 1.076 | | 1.013 | | 1.095 | |
| final *R* indices [*I*>2*σ*(*I*)] | *R*_1_ = 0.738  *wR*_2_ = 0.1794 | | *R*_1_ = 0.0401  *wR*_2_ = 0.1058 | | *R*_1_ = 0.0536  *wR*_2_ = 0.1358 | |
| *R* indices (all data) | *R*_1_ = 0.0862  *wR*_2_ = 0.1913 | | *R*_1_ = 0.0462  *wR*_2_ = 0.1087 | | *R*_1_ = 0.0694  *wR*_2_ = 0.1441 | |
| Largest diff peak/hole (e⋅Å^-3^) | 3.44/–0.58 | | 1.572/–1.355 | | 1.768/–1.798 | |
|  |  | |  | |  | |

**Table S5**. Crystal data and refinement of **7** and **8**

|  | **7** | | **8** | |
| --- | --- | --- | --- | --- |
| CCDC No. | 2227162 | 2227163 | |  |
| ormula | C_60_H_65_BiFeO_4_ | | C_61_H_65_BiCrO_5_ | |
| formula weight | 1114.95 | | 1139.11 | |
| crystal system | Orthorhombic | | Orthorhombic | |
| space group | *P*2_1_2_1_2_1_ | | *Pnma* | |
| *a*/Å | 11.2172(2) | | 19.6190(10) | |
| *b*/Å | 20.9094(5) | | 21.4772(12) | |
| c/Å | 23.8617(5) | | 12.9245(7) | |
| *α*/deg |  | |  | |
| *β*/deg |  | |  | |
| *γ*/deg |  | |  | |
| *V*/Å^3^ | 5596.6(2) | | 5445.9(5) | |
| *Z* | 4 | | 4 | |
| *ρ*_calcd_/g∙cm^-3^ | 1.323 | | 1.389 | |
| *μ*/mm^-1^ | 3.441 | | 5.547 | |
| *F*(000) | 2264 | | 2312 | |
| crystal size/mm^3^ | 0.24 x 0.22 x 0.20 | | 0.18 x 0.12 x 0.10 | |
| *θ* range/deg | 1.948–27.514 | | 3.988–53.897 | |
| collected data | 58496 | | 35408 | |
| unique data | 12803 (*R*_int_ = 0.0917) | | 5110  (*R*_int_ = 0.0516) | |
| GOF on *F*^2^ | 1.011 | | 1.056 | |
| final *R* indices [*I*>2*σ*(*I*)] | *R*_1_ = 0.0383  *wR*_2_ = 0.0847 | | *R*_1_ = 0.0443  *wR*_2_ = 0.1258 | |
| *R* indices (all data) | *R*_1_ = 0.0443  *wR*_2_ = 0.0873 | | *R*_1_ = 0.0581  *wR*_2_ = 0.1341 | |
| Largest diff peak/hole (e⋅Å^-3^) | 1.391/–0.844 | | 2.087/–1.130 | |

# Selected IR and NMR spectra


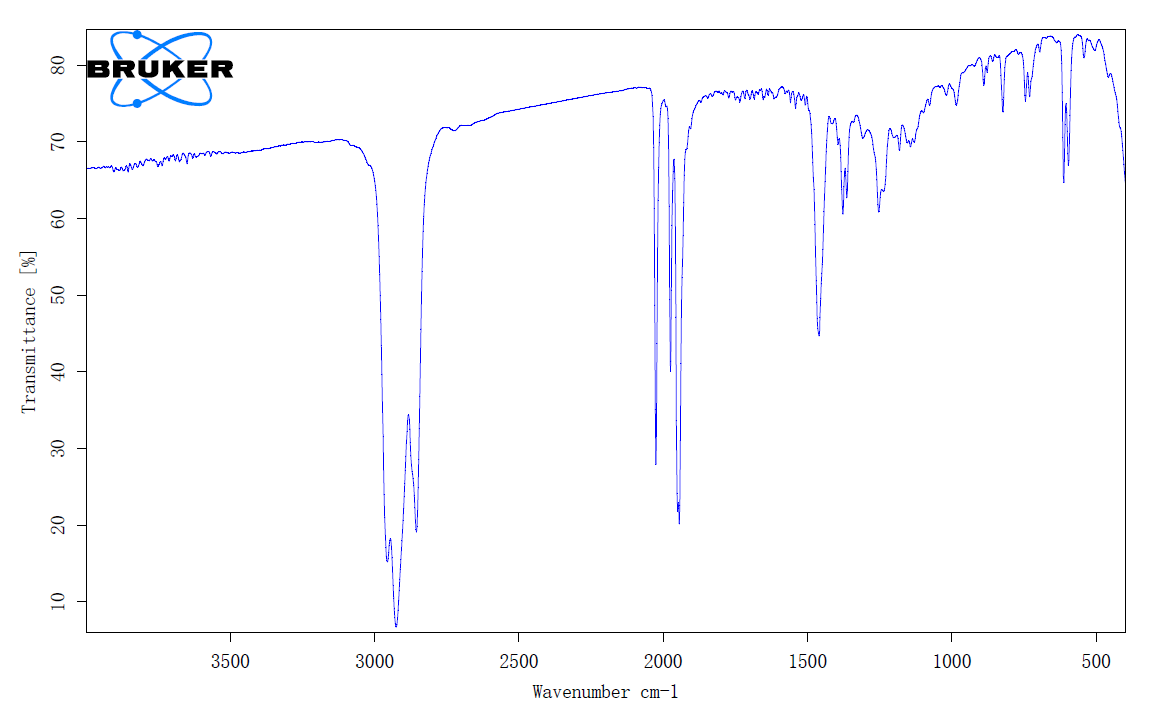


**Figure S17.** Infra-red absorption spectrum of **7** recorded in solid state at room temperature.


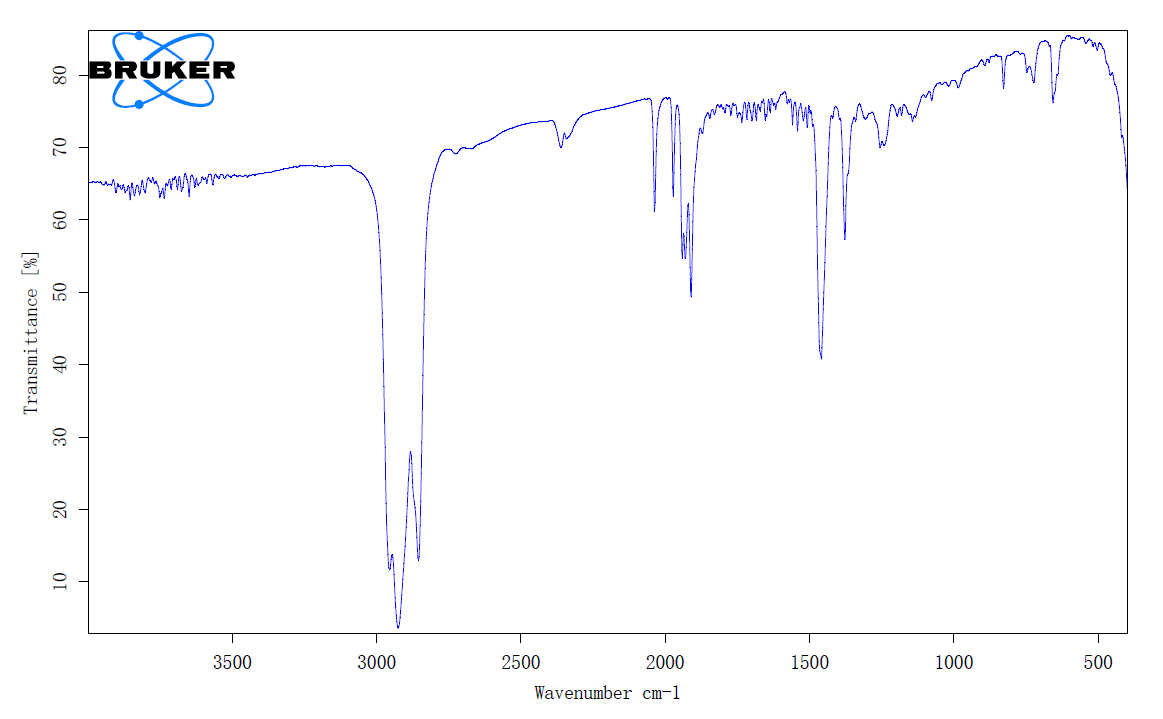


**Figure S18.** Infra-red absorption spectrum of **8** recorded in solid state at room temperature.

**Figure S19.** ^1^H NMR spectrum of **1** in CDCl_3_ at 298 K.

**Figure S20.** ^13^C{^1^H, ^13^C} NMR spectrum of **1** in CDCl_3_ at 298 K.

**Figure S21.** ^1^H NMR spectrum of **2** in CDCl_3_ at 298 K.

**Figure S22.** ^13^C{^1^H, ^13^C} NMR spectrum of **2** in CDCl_3_ at 298 K.

**Figure S23.** ^1^H NMR spectrum of **3** in THF-D_8_ at 298 K.

**Figure S24.** ^13^C{^1^H, ^13^C} NMR spectrum of **3** in THF-D_8_ at 298 K.

**Figure S25.** ^1^H NMR spectrum of **4** in THF-D_8_ at 298 K.

**Figure S26.** ^13^C{^1^H, ^13^C} NMR spectrum of **4** in THF-D_8_ at 298 K.

## Figure S27. ^1^H NMR spectrum of 5 in CDCl_3_ at 298 K.

## Figure S28. ^13^C{^1^H, ^13^C} NMR spectrum of 5 in CDCl_3_ at 298 K.

## Figure S29. ^1^H NMR spectrum of the NMR tube reaction of 3 and PhSSPh in C_6_D_6_ at 298 K, showing that there is no formation of H_2_. This suggests that there is no hydride at the bismuth atom in 3.

## Figure S30. ^1^H NMR spectrum of 6 in CDCl_3_ at 298 K.

## Figure S31. ^13^C{^1^H, ^13^C} NMR spectrum of 6 in CDCl_3_ at 298 K.

## Figure S32. ^1^H NMR spectrum of 7 in C_6_D_6_ at 298 K.

## Figure S33. ^13^C{^1^H, ^13^C} NMR spectrum of 7 in C_6_D_6_ at 298 K.

## Figure S34. ^1^H NMR spectrum of 8 in C_6_D_6_ at 298 K.

# Reference

1. He Y, Dai C, Wang D *et al.* Phosphine-Stabilized Germylidenylpnictinidenes as Synthetic Equivalents of Heavier Nitrile and Isocyanide in Cycloaddition Reactions with Alkynes. *J Am Chem Soc*. 2022; **144**(11): 5126–5135. doi: 10.1021/jacs.2c00305

2. Wang D, Zhai C, Chen Y *et al.* An isolable germylyne radical with a one-coordinate germanium atom. *Nat Chem*. 2022. doi: 10.1038/s41557-022-01081-1

3. Matthews SL, Heinekey DM. Photochemical Generation of Dihydrogen Complexes of Chromium and Tungsten. *J Am Chem Soc*. 2006; **128**(8): 2615-2620. doi: 10.1021/ja057912r

4. Sheldrick GM. Crystal structure refinement with SHELXL. *Acta Cryst*. 2015; **C71**: 3-8.

5. Dolomanov OV, Bourhis LJ, Gildea RJ *et al.* OLEX2: a complete structure solution, refinement and analysis program. *Journal of Applied Crystallography*. 2009; **42**(2): 339-341. doi: doi:10.1107/S0021889808042726

6. Spek A. PLATON SQUEEZE: a tool for the calculation of the disordered solvent contribution to the calculated structure factors. *Acta Cryst*. 2015; **C71**(1): 9-18. doi: doi:10.1107/S2053229614024929

7. Toma A, Raţ CI, Silvestru A *et al.* Organoantimony(III) and -bismuth(III) hypervalent pseudohalides. An experimental and theoretical study. *J Organomet Chem*. 2013; **745-746**: 71-79. doi: <https://doi.org/10.1016/j.jorganchem.2013.06.044>

8. Neese F. The ORCA program system. *WIREs Comput Mol Sci*. 2012; **2**(1): 73-78. doi: <https://doi.org/10.1002/wcms.81>

9. Douglas M, Kroll NM. Quantum electrodynamical corrections to the fine structure of helium. *Annals of Physics*. 1974; **82**(1): 89-155. doi: <https://doi.org/10.1016/0003-4916(74)90333-9>

10. Jansen G, Hess BA. Revision of the Douglas-Kroll transformation. *Phys Rev A*. 1989; **39**: 6016-6017. doi: 10.1103/PhysRevA.39.6016

11. Rolfes JD, Neese F, Pantazis DA. All-electron scalar relativistic basis sets for the elements Rb–Xe. *J Comput Chem*. 2020; **41**(20): 1842-1849. doi: <https://doi.org/10.1002/jcc.26355>

12. Johnson ER, Keinan S, Mori-Sánchez P *et al.* Revealing Noncovalent Interactions. *J Am Chem Soc*. 2010; **132**(18): 6498-6506. doi: 10.1021/ja100936w

13. Lu T, Chen F. Multiwfn: A multifunctional wavefunction analyzer. *J Comput Chem*. 2012; **33**(5): 580-592. doi: <https://doi.org/10.1002/jcc.22885>

14. Lu T, Chen Q. Interaction Region Indicator: A Simple Real Space Function Clearly Revealing Both Chemical Bonds and Weak Interactions**. *Chemistry–Methods*. 2021; **1**(5): 231-239. doi: <https://doi.org/10.1002/cmtd.202100007>

15. Roos BO. The Complete Active Space Self-Consistent Field Method and its Applications in Electronic Structure Calculations. *Advances in Chemical Physics*1987. 399-445.

16. Ruedenberg K, Cheung LM, Elbert ST. MCSCF optimization through combined use of natural orbitals and the brillouin–levy–berthier theorem. *Int J Quantum Chem*. 1979; **16**(5): 1069-1101. doi: <https://doi.org/10.1002/qua.560160511>

17. Roos BO, Taylor PR, Sigbahn PEM. A complete active space SCF method (CASSCF) using a density matrix formulated super-CI approach. *Chemical Physics*. 1980; **48**(2): 157-173. doi: <https://doi.org/10.1016/0301-0104(80)80045-0>

18. Angeli C, Cimiraglia R, Evangelisti S *et al.* Introduction of n-electron valence states for multireference perturbation theory. *J Chem Phys*. 2001; **114**(23): 10252-10264. doi: 10.1063/1.1361246

19. Grimme S, Ehrlich S, Goerigk L. Effect of the damping function in dispersion corrected density functional theory. *J Comput Chem*. 2011; **32**(7): 1456-1465. doi: <https://doi.org/10.1002/jcc.21759>

20. W. C. Martin, Table of Spin-Orbit Energies for p-Electrons in Neutral Atomic (core)np Configurations. J. Res. Natl. Bur. Stand. A Phys.Chem. 1971, **75**, 109-111.

21. Dolk L, Litzén U, Wahlgren GM. The laboratory analysis of Bi II and its application to the Bi-rich HgMn star HR 7775. *A&A*. 2002; **388**(2): 692-703.
